# Early stage of biofilm assembly on microplastics is structured by substrate size and bacterial motility

**DOI:** 10.1002/imt2.121

**Published:** 2023-06-07

**Authors:** Peng Qin, Han Cui, Panxin Li, Shuaitao Wang, Shen Fan, Jie Lu, Meng Sun, Heng Zhang, Shougang Wang, Xiaoyan Su, Hui‐Hui Fu, Xiaoli Hu, Jinshui Lin, Yu‐Zhong Zhang, Wei Ding, Weipeng Zhang

**Affiliations:** ^1^ Institute of Evolution & Marine Biodiversity Ocean University of China Qingdao China; ^2^ College of Marine Life Sciences Ocean University of China Qingdao China; ^3^ College of Life Sciences Yan'an University Yan'an China; ^4^ MOE Key Laboratory of Marine Genetics and Breeding Ocean University of China Qingdao China; ^5^ Frontiers Science Center for Deep Ocean Multispheres and Earth System, Ocean University of China Qingdao China; ^6^ State Key Laboratory of Microbial Technology Shandong University Qingdao China

## Abstract

The taxonomic structure of biofilms on 0.3‐mm microplastics differed significantly from that on 3‐mm microplastics or glass particles. Compared with the 3‐mm microplastics, biofilms on 0.3‐mm microplastics were enriched for genes involved in flagellar‐based motility and chemotaxis, pointing to a more ‘mobile’ community. The association between motility and bacterial colonization of 0.3‐mm microplastics was observed through laboratory experiments using isolated strains.

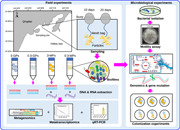

## INTRODUCTION

Microplastic particles (MPs), defined as plastic debris with diameters of less than 5 mm, are the dominant form of plastics in the oceans of the earth [1]. These have become one of the most severe threats to the entire marine ecosystem, largely due to poor biodegradation and harmful chemical compounds, many of which are persistent organic pollutants [2, 3, 4]. The most abundant type of marine MPs is polyethylene, followed by polypropylene, polyvinyl chloride, and polystyrene [5]. The hydrophobic surfaces of MPs allow bacterial colonization and biofilm formation. Such a biofilm on the MP surface is defined as a ‘plastisphere’, as first described during the study of MPs in the North Atlantic [6]. Thus, the ‘plastisphere’ represents a specific biofilm community, as marine biofilms can be found on nearly all substrates immersed in seawater, including animal body surfaces, rock surfaces, organic particles, and sediment–water interfaces [7]. Biofilm formation on MPs can, in turn, promote the spread of MPs [8]. First, biofilm formation can lead to increased MP density and, thus reduced buoyancy [8]. Second, the biofilm matrix is sticky and can promote the formation of heteroaggregates [8]. Biofilm formation on MPs can also impact element cycling in the marine ecosystem, and for example, the presence of MPs has been shown to alter nitrogen cycling processes [9]. These reports demonstrate the significance of MP‐associated microbiota and the environmental effects of MPs, indicating that the study of the mechanisms governing the assembly of biofilms on MPs is of great importance.

Various parameters are known to determine the taxonomic structures of MP‐associated biofilms [10, 11, 12, 13, 14, 15, 16]. For example, one study pointed out that the microbial diversity and uniformity in MP‐associated biofilms are higher than those in seawater, and they are co‐influenced by polymer types and exposure duration [11]. A study of environmental factors influencing the bacterial colonization of plastic debris suggested that seawater salinity is the main factor influencing microbial diversity in MP‐associated biofilms [12]. Interestingly, the color of the plastic was also found to affect both the taxonomic structure and functional composition of MP‐associated biofilms [13]. Moreover, the relationship between MP size (diameter) and the functional properties of MP‐associated biofilms has been investigated in a few studies [14, 15, 16]. For example, in wastewater treatment systems, the MP size influences the environmental behavior of antibiotic‐resistance genes [14]. However, the mechanisms governing the influence of MP size on bacterial colonization in in situ marine environments remain elusive.

In the present study, we conducted a biofilm formation experiment in a coastal marine area, by employing MPs and glass particles (GPs) of different sizes and immersing these materials in the subtidal zone for 10 and 20 days before recovery. Subsequently, metagenomics, 16S rRNA gene amplicon analyses, metatranscriptomics, and quantitative real‐time polymerase chain reaction (qRT‐PCR) were performed to elucidate the taxonomic and functional uniqueness of biofilms formed on MPs. Furthermore, experiments based on culturable bacterial strains were conducted to support our conclusion.

## RESULTS

### Taxonomic structures of biofilms on MPs and GPs, and seawater

**Figure 1:**
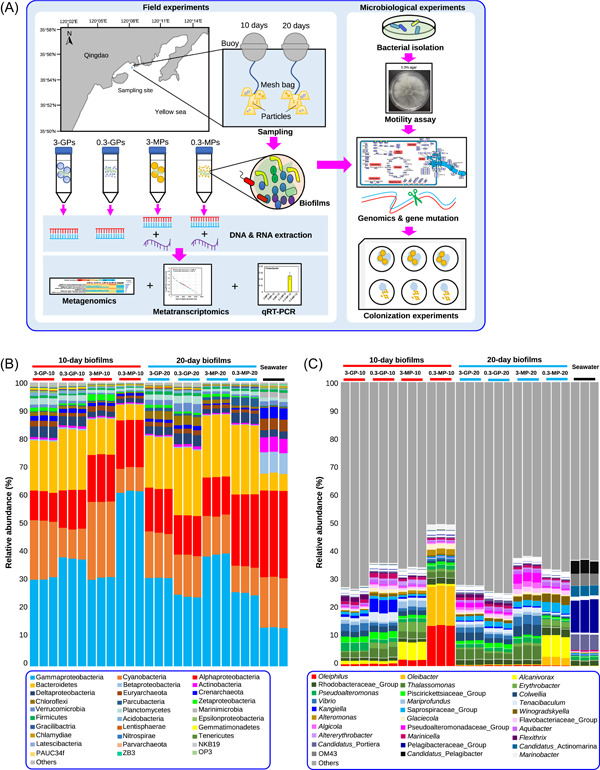
The experimental workflow and taxonomic composition of biofilm and seawater samples. (A) The experimental workflow. Biofilms were developed on two sizes (3 and 0.3 mm) of microplastic and glass particles, in the subtidal zone, for 10 and 20 days. Then they were transferred to the laboratory and subjected to metagenomic (three biological replicates) and metatranscriptomic sequencing and analyses. Experiments with an isolated bacterial strain were used to confirm the results of the *in silico* analyses. (B and C) Taxonomic composition analyses on miTags derived from the 27 metagenomes. The 30 most abundant phyla (B) or genera (C) in terms of maximum relative abundance are shown, and the remaining were merged into “Others.” Proteobacteria were classified down to the class level. The 10‐day biofilms on 3‐mm and 0.3‐mm glass particles, 3‐mm and 0.3‐mm microplastic particles were termed 3‐GP‐10, 0.3‐GP‐10, 3‐MP‐10, and 0.3‐MP‐10, respectively. The 20‐day biofilms were termed 3‐GP‐20, 0.3‐GP‐20, 3‐MP‐20, and 0.3‐MP‐20.

The experimental design is shown in Figure 1A. Before deploy into seawater, the surface characters of MPs were observed using scanning electron microscope (SEM), and no apparent difference could be identified (Figure S1). In total, 27 biofilm and seawater samples were collected and metagenomic sequencing of these samples generated a total of 554.09 Gb data sets with 20.52 ± 1.88 Gb per sample (Table S1). The 10‐day and 20‐day biofilms on 3‐mm MPs were termed 3‐MP‐10 and 3‐MP‐20, respectively. The 10‐day and 20‐day biofilms on 0.3‐mm MPs were termed 0.3‐MP‐10 and 0.3‐MP‐20, respectively. The biofilms on GPs were termed 3‐GP‐10, 3‐GP‐20, 0.3‐GP‐10, and 0.3‐GP‐20, following the same naming rule. Operational taxonomic unit (OTU) classification using miTags extracted from these metagenomes at 97% similarity revealed a total of 18,356 OTUs.

Taxonomic assignments of representative reads of the OTUs revealed a total of 78 phyla (Proteobacteria were further classified to the class level), and the phylum‐level community structures are displayed in Figure 1B. The taxonomic profiles of the seawater microbiota differed from those in the biofilms, with Alphaproteobacteria emerging as the most abundant taxa, while Betaproteobacteria, Actinobacteria, and Crenarchaeota made up a relatively larger proportion in comparison with their counterparts in the biofilms (Figure 1B). Gammaproteobacteria and Bacteroidetes were the most prevalent taxa across all the biofilms. Among all the biofilms assayed, 0.3‐MP‐10 was unique, with Gammaproteobacteria accounting for up to 61.57%, and fewer taxa were detected than in the other biofilms (Figure 1B). In addition, there appeared to be a structural transition from the 0.3‐MP‐10 to 0.3‐MP‐20 biofilms, indicated by the 3.0‐fold decline and 3.6‐fold rise of the relative abundances of Gammaproteobacteria and Bacteroidetes, respectively (Figure 1B). At the genus level, the taxonomic structure of the 0.3‐MP biofilms clearly differed from those of the other samples (Figure 1C). The most abundant genera, *Oleiphilus* and *Oleibacter*, accounted for averages of 14.71% and 14.35%, respectively, in the 0.3‐MP‐10 biofilms, while showing reduced abundance in other biofilms (Figure 1C). *Alteromonas* was also enriched in the 0.3‐MP‐10 biofilms, in comparison with other biofilms and the seawater microbiota. In addition, the structural transition from the 0.3‐MP‐10 to 0.3‐MP‐20 biofilms could also be observed at the genus level, as seen by the elimination of *Oleiphilus* during biofilm development from 10 to 20 days (Figure 1C). These results revealed that a unique taxonomic composition in earlier‐stage biofilms formed on 0.3‐MPs.

The dissimilarities between samples were then investigated using principal coordinate analysis (PCoA). The 10‐day and 20‐day‐immersed biofilms were analyzed separately using Bray‐Curtis and Jaccard distances. In all the analyses, an apparent boundary between the biofilms and the seawater samples was observed (Figure S2). In the PCoA of the 10‐day biofilm and the seawater samples, the 0.3‐MP‐10 biofilms were located separately from all the other biofilms (Figure S2). Similarly, in the PCoA of the 20‐day biofilm and the seawater samples, the 0.3‐MP‐20 biofilms were located separately from all the other biofilms (Figure S2). Overall, the PCoA plots revealed the uniqueness of the 0.3‐MP biofilms, especially 0.3‐MP‐10 biofilms, which is consistent with the taxonomic profiling results.

As the above analyses were based on miTags, we then used 16S rRNA amplicon (spanning the V3V4 regions) sequencing to verify the above results. Data information is given in Table S2. Each sample contained more than 50,000 amplicon sequences, and rarefaction curves based on the Chao1 and Shannon indexes indicated sequencing depth of sufficient richness and diversity (Figure S3). Consistent with the miTag results (Figure 1B), classification of the 16S rRNA gene amplicon sequences revealed the dominance of Gammaproteobacteria in the 0.3‐MP‐10 biofilms, with increases seen in the Bacteroidetes during 0.3‐MP biofilm development (Figure S4). At the genus level, *Oleiphilus*, *Oleibacter, Nautella*, and *Alteromonas* were enriched in the 0.3‐MP‐10 biofilms (Figure S5), also consistent with the miTags results (Figure 1C). Although PCoA based on the Jaccard dissimilarities could not separate the biofilms, PCoA based on Bray‐Curtis dissimilarities could clearly distinguish the 0.3‐MP‐10 biofilms from other 10‐day biofilms and the seawater microbiota (Figure S6), suggesting different taxonomic composition (identity and relative abundance). Together, these results derived from the 16S rRNA gene amplicon sequences have verified the miTags results.

### Enrichment of motility and chemotaxis genes in 0.3‐MP‐10 biofilms and their in situ expression

**Figure 2:**
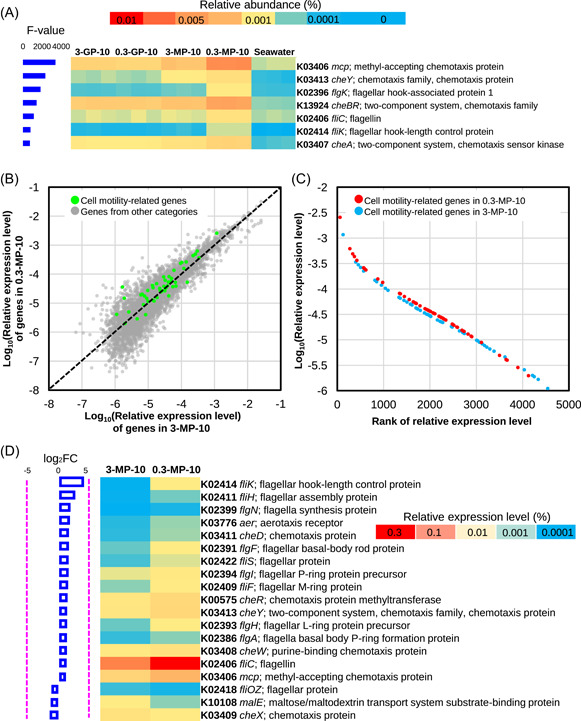
Metagenomics to reveal differentially presented genes of the 10‐day biofilms and metatranscriptomics to reveal expression levels of cell motility‐related genes in the 3‐MP‐10 and 0.3‐MP10 biofilms. (A) Annotation and abundance distribution of the top 7 (indicated by *F*‐value in one‐way analysis of variance [ANOVA]) significantly differentially presented motility‐related genes by metagenomics. The genes were annotated by the Kyoto Encyclopedia of Genes and Genomes (KEGG) and those in the 0.3‐MP‐10 biofilms were compared with those in the other four microbiota to identify significantly enriched genes (*p*‐value < 0.001 in one‐way ANOVA). (B) Relative abundance distribution of the total genes and cell motility‐related genes by metatranscriptomics. The relative abundance of genes was converted into an exponential form. (C) Expression‐level ranks of all cell motility‐related genes in the 0.3‐MP‐10 and 3‐MP10 biofilms by metatranscriptomics. The relative abundance of genes was also shown as an exponential form. (D) Functional profiles of the cell motility‐related genes. The genes are listed based on the fold change (FC = 0.3‐MP‐10/3‐MP‐10) of expression levels between the two biofilms revealed by metatranscriptomics.

The unique taxonomic structure in 0.3‐MP‐10 biofilms led us to further explore their functional specification. The 27 metagenomes were assembled individually, and then the open reading frames (ORFs) were predicted and annotated based on the Kyoto Encyclopedia of Genes and Genomes (KEGG) database. Of the 195,721 ± 47,617 predicted ORFs, 5493 ± 218 ORFs could be annotated (Table S1). After read mapping and summary, the relative abundance of each KEGG gene was revealed. Considering the taxonomic uniqueness of the 0.3‐MP‐10 biofilms, we used subsequent one‐way analysis of variance (ANOVA) to identify enriched KEGG genes in the 0.3‐MP‐10 biofilms by comparative analysis with the other four microbiota groups. We found that 3758 or the 6518 KEGG‐annotated genes were significantly changed (adjusted *p*‐value < 0.001 in one‐way ANOVA), and 749 were enriched in the 0.3‐MP‐10 biofilms. Classification of the 749 significantly enriched genes in the 0.3‐MP‐10 biofilms revealed a total of 20 functional categories, and motility was among the categories with the highest gene numbers (Figure S7). Of these significantly‐changed KEGG‐annotated genes, the top 40 genes, in terms of *F*‐value revealed by one‐way ANOVA, are shown in Figure S8. Notably, seven of these were related to flagellar biosynthesis and regulation, such as *flgK*: encoding the flagellar hook‐associated protein 1 (K02396) and *fliK*: encoding flagellar hook‐length control protein (K02414), and all these genes were enriched in the 0.3‐MP‐10 biofilms (Figure 2A).

The above functional analyses revealed the presence of a more “mobile” community during the early stages of biofilm development on the 0.3‐MPs. To confirm the result of one‐way ANOVA, we compared the KEGG genes in the 3‐MP‐10 biofilms with those in the 0.3‐MP‐10 biofilms using DESeq2 analysis, in which the reads number was used as queries. We found that 1460 KEGG‐annotated genes were significantly changed (adjusted *p*‐value < 0.001). Nearly all the flagellar‐related genes showed significant difference and enriched (fold change > 2) in the 0.3‐MP‐10 biofilms (Figure S9). For example, the *fliH* gene involved in flagellar assembly showed the largest fold change (Figure S9). Other abundant genes, such as *fliC*, *flgE*, and *motA*, were all enriched in 0.3‐MP‐10 biofilms, with more than two‐fold increase (Figure S9). In addition, we explored the taxonomic affiliations of *fliC* and *flgE* in 0.3‐MP‐10 biofilm metagenomes, after reads assembly, ORF prediction, and KEGG annotation. These genes were found to be most common in bacteria belonging to *Alteromonas*, *Cognatishimia*, and certain *Roseobacter* group members (e.g., *Sulfitobacter*, *Roseovarius*, and *Ruereria*) (Figure S10).

As the metagenomic comparison revealed that motility‐related genes were enriched in the 0.3‐MP‐10 biofilms, we used metatranscriptomics to examine the expression of these genes in the 3‐MP‐10 and the 0.3‐MP‐10 biofilms, with one metatranscriptome created for each. The basic information, including reads number and length, are provided in Table S3. The gene expression levels were shown by mapping the metatranscriptomic reads to the metagenome‐derived orthologs. The relative abundance of the overall gene expression profiles and expression‐level ranks are shown in Figure 2B,C, respectively. Distinct distribution patterns between the motility‐related genes and other genes were observed, with motility‐related genes tending to be located in the up‐left area and close to the y‐axis (representing 0.3‐MP‐10) while the other genes tended to be located in the bottom‐right area and close to the x‐axis (representing 3‐MP‐10) (Figure 2B). Consistently, rank analysis revealed higher expression levels of motility‐related genes in 0.3‐MP‐10 than in 3‐MP‐10 biofilms (Figure 2C). Then we took a closer look at the motility‐related genes in these two metatranscriptomes by profiling the KEGG genes individually (Figure 2D). Notably, most of these genes documented higher relative expression levels in the 0.3‐MP‐10 biofilm than in the 3‐MP‐10 biofilm, such as evident results seen for *fliK* (K02414), *flgF* (K02391), *fliC* (K02406), and *mcp* (K03406) (Figure 2D). In addition, we explored the taxonomic connection of *fliC* and *flgE* in 0.3‐MP‐10 to understand the active and mobile microbes (Figure S11). The 0.3‐MP‐10 metatranscriptome was assembled and subjected to ORF prediction and KEGG annotation for both function and taxonomy. Bacteria belonging to *Alteromonas*, *Pseudoalteronomas*, *Sulfitobacter*, *Thalassolituus*, and *Oleiphilus* were thus suggested to be the major hosts of the investigated genes (Figure S11).

To verify the metatranscriptomic results, we used qRT‐PCR to examine the expression of the *flgE* gene in 3‐MP and 0.3‐MP biofilms. Its expression in various bacterial taxa (*Alteromonas*, *Leisingera*, *Phaeobacter*, *Thalassolituus*, and *Vibrio*) and different stages (5, 10, and 15 days) of biofilm development was examined. The *flgE* gene was selected based on the taxonomic classification of metatranscriptome‐derived ORFs (respective primers are shown in Table S4). It was found that *flgE* was expressed in all five genera in the 0.3‐MP biofilms, but was only expressed in two genera (*Alteromonas* and *Vibrio*) in the 3‐MP biofilms (Figure S12). Moreover, a stage‐specific expression pattern was observed, as in the 0.3‐MP biofilms, *flgE* expression was only observed in 5 or 10 days but not in 15 days (Figure S12).

### Experimental evidence for the association between bacterial motility and colonization of 0.3‐MPs

The above analyses led us to hypothesize that there is an association between high motility and bacterial colonization of the 0.3‐MPs. We, therefore, conducted laboratory experiments using a single bacterial strain to test this hypothesis. We isolated strains from MPs that were immersed in seawater for one month and performed taxonomic identification for the selection of suitable strains for the experiments. A total of 350 stains (genus‐level affiliations are shown in Figure S13) were obtained and a strain belonging to *Alteromonas* was firstly selected as a candidate strain. Members of *Oleiphilus* or *Oleibacter* were also considered but difficulties in culturing these bacteria hindered subsequent experiments. The complete genome of the *Alteromonas* strain was sequenced. It was found to possess five rRNAs, 67 tRNAs, and 3743 ORFs, of which 1805 could be annotated by the KEGG database (Table S5). Whole‐genome searching against the GTDB‐Tk database classified this bacterium as *Alteromonas* sp009811495, and thus it was named *A*. sp009811495 PMMA93 (PMMA93 was the identity assigned in our bacterial culture). Analysis of its metabolic pathways with a focus on flagellar biosynthesis and energy metabolism revealed that the flagellar biosynthesis pathway was relatively complete, comprising about 34 genes encoding proteins associated with the motor, stator, basal body, proximal rod, rings, hook, filament, and cap (Figure S14). The genome possessed a complete Embden‐Meyerhof‐Parnas pathway and all genes for the tricarboxylic acid cycle (Figure S14), suggesting that this bacterium was a heterotrophic organism. Genes for nitrate and nitrite respiration were also annotated (Figure S14), suggesting its potential for living without oxygen. Moreover, a number of genes responsible for biofilm formation and chemotaxis, as well as relevant regulatory genes, were identified (Figure S14). In addition, this bacterium possessed a complete cell membrane‐supported respiratory chain (Figure S14), which can provide the energy required for swimming and swarming. To confirm the motility ability of PMMA93, we performed a motility assay on marine broth 2216E agar plates. Another isolated strain, *Polaribacter dokdonensis* PC73 was used as a negative control (the genus *Polaribacter* was not detected in 0.3‐MP‐10 biofilm metagenomes). On the plates with 0.3% (w/v) agar, PMMA93 occupied almost the whole plate area after swimming for 5 days at 25°C, whereas PC73 showed no sign of motility (Figure S15). On the plates with 0.5% (w/v) agar, PMMA93 was found to swarm after 5 days, indicated by a loose colony with a diameter of 2.7 cm (Figure S15).

**Figure 3:**
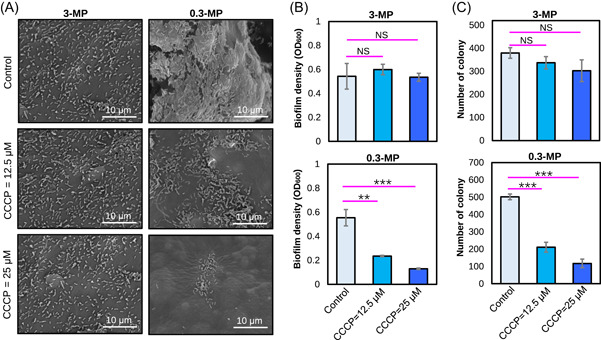
Biofilm formation of *Alteromonas* sp009811495 PMMA93 treated with different concentrations of carbonyl cyanide 3‐chlorophenylhydrazone (CCCP). PMMA93 cells were incubated with 3‐MP or 0.3‐MP together with the addition of 0, 12.5, and 25 μm CCCP for 12 h, followed by scanning electron microscope evaluation and imaging (A), cell density measurement (B), and colony counting (C). The CCCP treatment was found to exert a dose‐dependent impact on PMMA93 colonization on the 0.3‐MP while having no significant effect on its colonization on the 3‐MP. The error bars represent the standard deviation of three biological replicates. ** indicates *p*‐value < 0.01, *** indicates *p*‐value < 0.001, while NS indicates nonsignificant.

To assess the contribution of motility on biofilm formation on microplastics, we cultured bacterial cells of PMMA93 at 25°C up to the log phase, treated them with different concentrations of carbonyl cyanide 3‐chlorophenylhydrazone (CCCP), a compound that can eliminate proton motive force (PMF), and then incubated them with the 3‐MPs and 0.3‐MPs used in the metagenomic study. After 12 h, bacterial cells colonized on the MPs were washed out and subjected to SEM observation (Figure 3A), optical density measurement at 600 nm (OD_600_) (Figure 3B), and the counting of colonies plated on agar media (Figure 3C). The CCCP treatment exerted a dose‐dependent impact on bacterial colonization on the 0.3‐MPs with 12.5 µΜ and 25 µΜ of CCCP abrogating its colonization by over 50% and about 75% of cells, respectively (Figure 3A–C). In contrast, no significant impact of CCCP on bacterial colonization of 3‐MPs could be detected (Figure 3A–C). To further investigate bacterial colonization of MPs, we conducted a coculture experiment using a motile strain (*A*. sp009811495 PMMA93) and a nonmotile strain (*Polaribacter* sp. PC73). Cells of these two strains were mixed in a 1:1 ratio and incubated with 0.3‐MP or 3‐MP at 25°C for 12 h. This was followed by 16S rRNA gene amplicon sequencing to investigate their relative abundance in the biofilm‐associated (cells colonized the MPs) and the free‐living (in the media) phases. Information on the 16S rRNA gene sequences is shown in Table S6. Subsequent analysis revealed that PC73 accounted for approximately 50% in the free‐living phases, regardless of the MP size (Figure S16A). However, the percentage of PC73 decreased to 29%–37% in cells colonized 3‐MPs and to 10%–15% in cells colonized 0.3‐MPs (Figure S16A). Statistical analysis revealed a significantly (*t*‐test, *p*‐value < 0.01) higher percentage of PC73 on 3‐MPs than on 0.3‐MPs (Figure S16B). In contrast, the highest percentage of PMMA93 was observed in biofilms on 0.3‐MPs (Figure S16A,B).

The above results indicated that the mobile strain (*A*. sp009811495 PMMA93) colonize 0.3‐MPs more effectively. To confirm this finding, we examined the impact of CCCP on the colonization of MPs by other mobile strains. Three strains isolated from marine MPs, including *Vibrio alginolyticus* 2‐8, *Stentrophomonas pavanii* P5G5, and *Tritonibacter mobilis* 2‐12, were used for this experiment. Similar to PMMA93, colonization of 0.3‐MP by these three strains was largely abolished by CCCP, whereas the impact of CCCP on their colonization of 3‐MPs was relatively slight, as indicated by different fold changes of the cell numbers (Figure S17). Finally, to further confirm the correlation between motility and bacterial colonization, we knocked out the *motAB* genes (primer sequences are given in Table S7) within a lateral flagellar gene cluster in *V. alginolyticus* 2–8 and compared the colonizing ability of the mutant with the wild‐type strain. The mutant displayed a significant defect in colonizing 0.3‐MPs, whereas no significant difference was detected between the cell number of the wild‐type strain on 3‐MPs and that of the mutant (Figure S18).

## DISCUSSION

In the present study, we investigated the influence of microplastic size on microbial colonization and marine biofilm formation by immersing microplastic and glass particles of different sizes in the subtidal zone. Based on miTags and 16S rRNA gene amplicon analyses, we showed that biofilm formed on the 0.3‐MPs, especially in the earlier stage (10‐day development), had unique taxonomic and functional structures. The enrichment of motility‐related genes motivated us to perform laboratory experiments using isolated strains to demonstrate the association between bacterial motility and successful colonization on 0.3‐MPs.

It has been suggested that the plastisphere is “just” a normal biofilm, which is not special in comparison with biofilms on other surfaces (e.g., subtidal stone surfaces) [17]. This view can be supported by the finding that differences between microbial communities on MPs and other materials are usually driven by rare taxa while abundant groups remain unchanged [18, 19], and environmental variables often have a much larger influence on the community structure than MP type [20, 21]. In contrast, here we demonstrated the taxonomic uniqueness of biofilms on the 0.3‐MPs, especially in their earlier stage, which was largely due to abundant genera represented by *Oleiphilus* and *Oleibacter*. *Oleiphilus* has been found to form biofilm on the surfaces of oil droplets [22] while *Oleibacter* contains giant flagellins that form thick flagellar filaments, which were speculated to facilitate adaptation to specific niches [23]. Therefore, the presence of bacteria in these two genera is consistent with our functional analyses, which highlighted the enrichment of motility‐related genes in the 0.3‐MP‐10 biofilms. Moreover, the 0.3‐MP‐20 biofilms were structurally different compared with the 0.3‐MP‐10 biofilms, which can be explained by the increasing influence of intra‐community forces during the biofilm development. Similar observations have been documented in previous studies on biofilm development [24, 25, 26]. For example, in the subtidal zone, the age of the biofilm was found to have a stronger effect in shaping the biofilm community structure than the substrate [25]. These observations imply that such intra‐community forces might involve species‐species interactions that reduce the effects of the biofilm substrate on microbial taxa, and such interactions can result in the replacement of certain pioneer species (e.g., *Oleiphilus*). In addition, the results from the miTags and 16S rRNA gene amplicon analyses are largely consistent, suggesting that both approaches are suitable to address the taxonomic characteristics of the plastisphere.

Both metagenomics and metatranscriptomica indicated relatively “mobile” communities on 0.3‐MPs immersed in seawater for 10 days. Mechanistically, the enrichment of motility‐related genes can be explained by the physical features of 0.3‐MP surfaces, as attachment to these materials encounters stronger hydrodynamic forces compared with other materials when immersed in water. Hydrodynamic forces arising from water velocity and Brownian movement of the suspended small particles in water can be partially arrested by bacterial swimming, which provides a “kinetic force” to counteract the hydrodynamic force, while also preventing the attachment of nonmobile cells [27]. This explains the well‐known capability of *Oleiphilus* and *Oleibacter* to degrade and consume oil as they require increased motility to overcome hydrostatic forces at oil‐seawater interfaces. This mechanism is also consistent with a recent study in which motility enhanced the adhesion of a *Halomonas* strain to surfactant‐coated oil droplets dispersed in artificial seawater [28]. Moreover, unlike many particles, MPs can be electrostatically charged [29], which may increase the difficulty of bacterial colonization and cause the difference in the microbial composition between MPs and GPs, as several motility‐related genes showed higher abundance in 0.3‐MP‐10 and 3‐MP‐10 when compared with 0.3‐GP‐10 and 3‐GP‐10 biofilms. In addition, the larger specific surface areas of the 0.3‐MPs may contribute to the attachment of mobile bacteria as these bacteria have a greater chance of contacting these surfaces in a confined space, such as in the bag containing the particles during experimentation in the present study. Besides these physical mechanisms, the prevalence of chemotaxis‐related genes implies a possibility that compounds released from the MPs can play roles in recruiting particular bacterial species. For example, additives and oligopolymers can be released from MPs into water [30] and are likely to be consumed by certain microbial taxa. This notion is partially in line with the major conclusion of a previous study [31], in which the authors immersed chemically defined particles in coastal seawater and found that both motility and the ability of particle consumption contributed to microbial community assembly. These communities underwent rapid turnover, developing from a community capable of degrading the carbon particles to one that could not within 140 h [31], and it is likely to be the same case in the current study. However, the roles of MP‐released chemicals and their contribution to biofilm assembly need to be further studied.

To support and corroborate the field experiments, we used microbiological experiments on four isolated representative strains. Consistent with the results of the field experiments, the laboratory experiments on these bacterial strains highlighted the importance of motility during 0.3‐MP colonization. Because bacterial motility is driven by PMFs [32], our experiments also suggest that the colonization is probably an energy‐consuming process, consistent with the previous observation that flagellar motility is a complex cellular process that requires high energy investment for host colonization [33]. Moreover, using a coculture experiment including one mobile and one nonmobile strain, we suggested that motility is likely to facilitate greater competitiveness during bacterial colonization of 0.3‐MPs, and this finding is also in line with the previous understanding that bacterial motility leads to greater competitive advantage when colonizing nutrient patches [34]. The higher abundance of the mobile strain on 0.3‐MPs after 12 h of cultivation also implies a faster propagation rate after successful colonization. Finally, mutation of *motAB* within the lateral flagellar gene cluster, which is specifically involved in *Vibrio* swarming over surfaces rather than in liquid medium [35], further confirmed the role of motility in colonizing 0.3‐MPs. Together, these findings suggest that colonization of 0.3‐MPs requires stronger motility is likely to be common to many bacterial taxa.

There are several limitations to this study. First, only one location was used for biofilm development and the influences of environmental fluctuations were not considered. Although the effects of environmental parameters on microbial community composition of the plastisphere have been documented in several previous studies [10, 12], the underlying functional basis is not well understood, and this would be one of our future directions. Moreover, only two stages of biofilm development were selected for metagenomic and metatranscriptomic analyses. To complement this limitation, qRT‐PCR was conducted, which showed that the expression of motility‐related genes was higher in 0.3‐MP biofilms grown for 5 and 10 days than that in the 3‐MP biofilms. In addition, only four of the isolated strains were selected to support the results of the field experiment. To overcome this shortcoming and further our understanding of marine biofilm development, we are currently attempting the construction of a synthetic community comprising hundreds of isolated strains, which would be used as a model community to decipher the strain‐ and single‐gene level mechanisms for marine biofilm assembly.

## CONCLUSION

Combining the results of meta‐analyses and microbiological experiments, we propose that biofilm assembly on microplastics is structured by the association between substrate size and bacterial motility, which creates unique microbial communities. These findings have further refreshed our understanding of biofilm assembly and microbe‐surface interactions that possibly underpin the ecological roles and transport of microplastics in marine environments.

## AUTHOR CONTRIBUTIONS

Weipeng Zhang and Wei Ding conceived the project. Peng Qin and Han Cui performed the major part of the data analyses and experiments. Jinshui Lin, Panxin Li, and Shuaitao Wang performed the gene mutation experiment. Shen Fan, Jie Lu, Meng Sun, Heng Zhang, Shougang Wang, and Xiaoyan Su were involved in data analyses or experiments. Yu‐Zhong Zhang, Xiaoli Hu, and Hui‐Hui Fu provided technical support and comments. Weipeng Zhang and Peng Qin wrote the manuscript.

## CONFLICT OF INTEREST STATEMENT

The authors declare no conflict of interest.

## Supporting information

Supporting information.

Supporting information.

## Data Availability

New sequencing data was used in this article. The metagenomic, 16S rRNA gene amplicon, and metatranscriptomic data sets were uploaded to NCBI under the accession number PRJNA902427 (https://www.ncbi.nlm.nih.gov/sra/PRJNA902427). The complete genome of *Alteromonas* sp009811495 PMMA93 was uploaded to NCBI under the accession number SAMN32034847 (https://www.ncbi.nlm.nih.gov/nuccore/CP113972). Supplementary materials (methods, figures, tables, graphical abstract, Chinese translated version and update materials) may be found in the online DOI or iMeta Science http://www.imeta.science/.

## References

[1] Auta, Helen S. , Emenike Chijioke , and Shahul H. Fauziah . 2017. “Distribution and Importance of Microplastics in the Marine Environment: A Review of the Sources, Fate, Effects, and Potential Solutions.” Environment International 102: 165–76. 10.1016/j.envint.2017.02.013 28284818

[2] Hale, Robert C. , Meredith E. Seeley , Mark J. L. Guardia , Lei Mai , and Eddy Y. Zeng . 2020. “A Global Perspective on Microplastics.” Journal of Geophysical Research: Oceans 125: e2018JC014719. 10.1029/2018JC014719

[3] Teuten, Emma L. , Jovita M. Saquing , Detlef R. U. Knappe , Morton A. Barlaz , Susanne Jonsson , Annika Björn , Steven J. Rowland , et al. 2009. “Transport and Release of Chemicals from Plastics to the Environment and to Wildlife.” Philosophical Transactions of the Royal Society B: Biological Sciences 364: 2027–45. 10.1098/rstb.2008.0284 PMC2873017 19528054

[4] Rios, Lorena M. , Patrick R. Jones , Charles Moore , and Urja V. Narayan . 2010. “Quantitation of Persistent Organic Pollutants Adsorbed on Plastic Debris from the Northern Pacific Gyre's “Eastern Garbage Patch”.” Journal of Environmental Monitoring 12: 2226–36. 10.1039/c0em00239a 21042605

[5] Suaria, Giuseppe , Carlo G. Avio , Annabella Mineo , Gwendolyn L. Lattin , Marcello G. Magaldi , Genuario Belmonte , Charles J. Moore , Francesco Regoli , and Stefano Aliani . 2016. “The Mediterranean Plastic Soup: Synthetic Polymers in Mediterranean Surface Waters.” Scientific Reports 6: 37551. 10.1038/srep37551 27876837 PMC5120331

[6] Zettler, Erik R. , Tracy J. Mincer , and Linda A. Amaral‐Zettler . 2013. “Life in the “Plastisphere”: Microbial Communities on Plastic Marine Debris.” Environmental Science & Technology 47: 7137–46. 10.1021/es401288x 23745679

[7] Flemming, Hans‐Curt , and Stefan Wuertz . 2019. “Bacteria and Archaea on Earth and their Abundance in Biofilms.” Nature Reviews Microbiology 17: 247–60. 10.1038/s41579-019-0158-9 30760902

[8] Rummel, Christoph D. , Annika Jahnke , Elena Gorokhova , Dana Kühnel , and Mechthild Schmitt‐Jansen . 2017. “Impacts of Biofilm Formation on the Fate and Potential Effects of Microplastic in the Aquatic Environment.” Environmental Science & Technology Letters 4: 258–67. 10.1021/acs.estlett.7b00164

[9] Seeley, Meredith E. , Bongkeun Song , Renia Passie , and Robert C. Hale . 2020. “Microplastics Affect Sedimentary Microbial Communities and Nitrogen Cycling.” Nature Communications 11: 2372. 10.1038/s41467-020-16235-3 PMC7217880 32398678

[10] Miao, Lingzhan , Yue Yu , Tanveer M. Adyel , Chengqian Wang , Zhilin Liu , Songqi Liu , Liuyan Huang , et al. 2021. “Distinct Microbial Metabolic Activities of Biofilms Colonizing Microplastics in Three Freshwater Ecosystems.” Journal of Hazardous Materials 403: 123577. 10.1016/j.jhazmat.2020.123577 32795819

[11] Zhang, Sheng‐Jie , Yan‐Hua Zeng , Jian‐Ming Zhu , Zhong‐Hua Cai , and Jin Zhou . 2022. “The Structure and Assembly Mechanisms of Plastisphere Microbial Community in Natural Marine Environment.” Journal of Hazardous Materials 421: 126780. 10.1016/j.jhazmat.2021.126780 34358974

[12] Li, Wenjie , Ying Zhang , Nan Wu , Ze Zhao , Wei'an Xu , Yongzheng Ma , and Zhiguang Niu . 2019. “Colonization Characteristics of Bacterial Communities on Plastic Debris Influenced by Environmental Factors and Polymer Types in the Haihe Estuary of Bohai Bay, China.” Environmental Science & Technology 53: 10763–73. 10.1021/acs.est.9b03659 31441645

[13] Wen, Bin , Jun‐Heng Liu , Yuan Zhang , Hao‐Ran Zhang , Jian‐Zhong Gao , and Zai‐Zhong Chen . 2020. “Community Structure and Functional Diversity of the Plastisphere in Aquaculture Waters: Does Plastic Color Matter? Science of the Total Environment 740: 140082. 10.1016/j.scitotenv.2020.140082 32927571

[14] Wang, Zhufang , Yinglong Su , Jundong Zhu , Dong Wu , and Bing Xie . 2022. “Size‐Dependent Effects of Microplastics on Antibiotic Resistance Genes Fate in Wastewater Treatment Systems: The Role of Changed Surface Property and Microbial Assemblages in a Continuous Exposure Mode.” Science of the Total Environment 851: 158264. 10.1016/j.scitotenv.2022.158264 36037899

[15] Lee, Jongkeun , Seulki Jeong , Chenghua Long , and Kartik Chandran . 2022. “Size Dependent Impacts of a Model Microplastic on Nitrification Induced by Interaction with Nitrifying Bacteria.” Journal of Hazardous Materials 424: 127363. 10.1016/j.jhazmat.2021.127363 34634706

[16] Yang, Xiangyu , Lu Zhang , Yi Chen , Qiang He , Tao Liu , Guoqing Zhang , Ling Yuan , et al. 2022. “Micro (nano) Plastic Size and Concentration Co‐Differentiate Nitrogen Transformation, Microbiota Dynamics, and Assembly Patterns in Constructed Wetlands.” Water Research 220: 118636. 10.1016/j.watres.2022.118636 35623147

[17] Wright, Robyn J. , Gabriel Erni‐Cassola , Vinko Zadjelovic , Mira Latva , and Joseph A. Christie‐Oleza . 2020. “Marine Plastic Debris: A New Surface for Microbial Colonization.” Environmental Science & Technology 54: 11657–72. 10.1021/acs.est.0c02305 32886491

[18] Kirstein, Inga V. , Antje Wichels , Georg Krohne , and Gunnar Gerdts . 2018. “Mature Biofilm Communities on Synthetic Polymers in Seawater‐Specific or General?” Marine Environmental Research 142: 147–54. 10.1016/j.marenvres.2018.09.028 30337052

[19] Kirstein, Inga Vanessa , Antje Wichels , Elisabeth Gullans , Georg Krohne , and Gunnar Gerdts . 2019. “The Plastisphere‐Uncovering Tightly Attached Plastic “Specific” Microorganisms.” PLoS One 14: e0215859. 10.1371/journal.pone.0215859 31013334 PMC6478340

[20] Kettner, Marie Therese , Sonja Oberbeckmann , Matthias Labrenz , and Hans‐Peter Grossart . 2019. “The Eukaryotic Life on Microplastics in Brackish Ecosystems.” Frontiers in Microbiology 10: 538. 10.3389/fmicb.2019.00538 30949147 PMC6435590

[21] Kesy, Katharina , Sonja Oberbeckmann , Bernd Kreikemeyer , and Matthias Labrenz . 2019. “Spatial Environmental Heterogeneity Determines Young Biofilm Assemblages on Microplastics in Baltic Sea Mesocosms.” Frontiers in Microbiology 10: 1–18. 10.3389/fmicb.2019.01665 31447791 PMC6696623

[22] Golyshin, Peter N. , Tatiana N. Chernikova , Wolf‐Rainer Abraham , Heinrich Lünsdorf , Kenneth N. Timmis , and Michail M. Yakimov . 2002. “ *Oleiphilaceae* Fam. Nov., to Include *Oleiphilus messinensis* Gen. Nov., Sp. Nov., a Novel Marine Bacterium that Obligately Utilizes Hydrocarbons.” International Journal of Systematic and Evolutionary Microbiology 52: 901–11. 10.1099/00207713-52-3-901 12054256

[23] Thomson, Nicholas M. , Josie L. Ferreira , Teige R. Matthews‐Palmer , Morgan Beeby , and Mark J. Pallen . 2018. “Giant Flagellins Form Thick Flagellar Filaments in Two Species of Marine γ‐Proteobacteria.” PLoS One 13: e0206544. 10.1371/journal.pone.0206544 30462661 PMC6248924

[24] Zhang, Weipeng , Yong Wang , Ren Mao Tian , Salim Bougouffa , Bo Yang , Hui Luo Cao , Gen Zhang , et al. 2014. “Species Sorting during Biofilm Assembly by Artificial Substrates Deployed in a Cold Seep System.” Scientific Reports 4: 6647. 10.1038/srep06647 25323200 PMC4200420

[25] Chung, Hong Chun , On On Lee , Yi‐Li Huang , Siu Yan Mok , Roberto Kolter , and Pei‐Yuan Qian . 2010. “Bacterial Community Succession and Chemical Profiles of Subtidal Biofilms in Relation to Larval Settlement of the Polychaete *Hydroides elegans* .” The ISME Journal 4: 817–28. 10.1038/ismej.2009.157 20090788

[26] Huang, Yi‐Li , Jang‐Seu Ki , On On Lee , and Pei‐Yuan Qian . 2009. “Evidence for the Dynamics of Acyl Homoserine Lactone and AHL‐Producing Bacteria During Subtidal Biofilm Formation.” The ISME Journal 3: 296–304. 10.1038/ismej.2008.105 18987676

[27] Kerchove, Alexis J. , and Menachem Elimelech . 2008. “Bacterial Swimming Motility Enhances Cell Deposition and Surface Coverage.” Environmental Science & Technology 42: 4371–77. 10.1021/es703028u 18605557

[28] Dewangan, Narendra K. , and Jacinta C. Conrad . 2020. “Bacterial Motility Enhances Adhesion to Oil Droplets.” Soft Matter 16: 8237–44. 10.1039/d0sm00944j 32935718

[29] Seidensticker, Sven , Peter Grathwohl , Jonas Lamprecht , and Christiane Zarfl . 2018. “A Combined Experimental and Modeling Study to Evaluate Ph‐Dependent Sorption of Polar and Non‐Polar Compounds to Polyethylene and Polystyrene Microplastics.” Environmental Sciences Europe 30: 30. https://enveurope.springeropen.com/articles/10.1186/s12302-018-0155-z 30148026 10.1186/s12302-018-0155-z PMC6096972

[30] Bandow, Nicole , Verena Will , Volker Wachtendorf , and Franz‐Georg Simon . 2017. “Contaminant Release from Aged Microplastic.” Environmental Chemistry 14: 394–405. 10.1071/EN17064

[31] Datta, Manoshi S. , Elzbieta Sliwerska , Jeff Gore , Martin F. Polz , and Otto X. Cordero . 2016. “Microbial Interactions Lead to Rapid Micro‐Scale Successions on Model Marine Particles.” Nature Communications 7: 11965. 10.1038/ncomms11965 PMC4915023 27311813

[32] Manson, M. D. , P. Tedesco , H. C. Berg , F. M. Harold , and C. Van der Drift . 1977. “A Proton Motive Force Drives Bacterial Flagella.” Proceedings of the National Academy of Sciences 74: 3060–64. 10.1073/pnas.74.7.3060 PMC431412 19741

[33] Khan, Fazlurrahman , Nazia Tabassum , Raksha Anand , and Young‐Mog Kim . 2020. “Motility of *Vibrio* spp.: Regulation and Controlling Strategies.” Applied Microbiology and Biotechnology 104: 8187–208. 10.1007/s00253-020-10794-7 32816086

[34] Gude, Sebastian , Erçağ Pinçe , Katja M. Taute , Anne‐Bart Seinen , Thomas S. Shimizu , and Sander J. Tans . 2020. “Bacterial Coexistence Driven by Motility and Spatial Competition.” Nature 578: 588–92. 10.1038/s41586-020-2033-2 32076271

[35] Atsumi, Tatsuo , Linda McCartert , and Yasuo Imae . 1992. “Polar and Lateral Flagellar Motors of Marine *Vibrio* Are Driven by Different Ion‐Motive Forces.” Nature 355: 182–84. 10.1038/355182a0 1309599

