# Supplementary material for: Early stage of biofilm assembly on microplastics is structured by substrate size and bacterial motility
Source: Imeta. 2023 Jun 7;2(3):e121. doi: 10.1002/imt2.121 (PMC10989967; doi:10.1002/imt2.121)
Supplement: Supplementary file 1 — Supporting information. [file IMT2-2-e121-s001.docx]

**Supporting information to:**

**Early stage of biofilm assembly on microplastics is structured by substrate size and bacterial motility**

Running title: Marine biofilm assembly on microplastics

Peng Qin^1,2,#^ | Han Cui^1,2,#^ | Panxin Li^3^ | Shuaitao Wang^1,2^ | Shen Fan^1,2^ | Jie Lu^1,2^ | Meng Sun^1,2^ | Heng Zhang^1,2^ |

Shougang Wang^2,4^ | Xiaoyan Su^2,5^ | Hui-Hui Fu^2,5^ | Xiaoli Hu^2,4^ | Jinshui Lin^3^ | Yu-Zhong Zhang^2,5,6^ |

Wei Ding^2,4,*^ | Weipeng Zhang^1,2,*^

^1^ Institute of Evolution & Marine Biodiversity, Ocean University of China, Qingdao, China

^2^ College of Marine Life Sciences, Ocean University of China, Qingdao, China

^3^ College of Life Sciences, Yan’an University, Yan’an, China

^4^ MOE Key Laboratory of Marine Genetics and Breeding, Ocean University of China, Qingdao, China

^5^ Frontiers Science Center for Deep Ocean Multispheres and Earth System, Ocean University of China, Qingdao, China

^6^ State Key Laboratory of Microbial Technology, Shandong University, Qingdao, China

^#^ These authors have equal contribution

* Correspondence to Wei Ding ([dingwei@ouc.edu.cn](mailto:dingwei@ouc.edu.cn)) or Weipeng Zhang ([zhangweipeng@ouc.edu.cn](mailto:zhangweipeng@ouc.edu.cn))

**METHODS**

**Materials**

Polyethylene, being one of the most common types of MPs in the ocean, was chosen for experiments in the current study, and GP was set as a reference. To elaborate, two sizes of MP and GP, 3 mm corresponding to the mm grade and 0.3 mm corresponding to the μm grade, were used. The density of the MP was 0.94-0.96 g/cm^3^ and the density of the GP was 2.5 g/cm^3^. Both the MP and GP materials were purchased from Ruixiang Plasticizing Co., Ltd. (Guangzhou, China). Nylon mesh bags (mesh size = 74 μm, bag width = 20 cm, and bag height = 30 cm; Sangon Biotech, Shanghai, China) were used to hold the particles, which accounted for one-fifth of the bag volume. This setting allowed a free exchange of seawater while preventing the escape of particles. The particles and nylon mesh bags were washed three times with sterile seawater and then sterilized by autoclaving.

**Biofilm development and sample preparation**

Considering that biofilm development in the marine environment has different stages [25,36], we collected 10-day and 20-day biofilms formed on MPs and GPs with two different sizes. During the experimental period (October 2021 to November 2021), the four types of substrates were placed in the nylon mesh bags and immersed in the subtidal zone of the DingjiaTsui of Huangdao, the Yellow Sea (120^◦^8’19’’E, 35^◦^54’55’’N), to allow biofilm development for 10 and 20 days. Each type of substrate had three replicates and the bags were suspended in seawater at a 2-m depth using buoys. In addition, three seawater samples (5 L/sample) were collected at a depth of 2 m over the experimental period, and thus there were a total of 27 samples. After transfer to the laboratory, the MPs and GPs were rinsed three times with sterile seawater to remove loosely attached cells. After ultrasonication and vortexing for 5 min, the bacteria attached to the particles were collected in sterile seawater. Half of the collected bacterial cell sample was used for DNA extraction and the remaining sample was frozen in liquid nitrogen for RNA extraction. The seawater samples were filtered through a 0.1-mm plankton net to remove impurities and then filtered through 0.22-μm membranes fixed in a bore diameter filter. The collected membranes were used for DNA extraction.

**SEM observation**

MPs with or without colonized bacterial cells were soaked in 5% glutaraldehyde for cell fixation and then dehydrated with a 30-100% ethanol gradient for 10 min before being critical-point-dried with CO_2_. Dried specimens were sputter-coated with gold and platinum (10 nm) for 5 min using a Hitachi MC1000 Ion Sputter (Japan). Observations were done using a field emission SEM (Hitachi S-3400 N, Japan) operated at an accelerating voltage of 5 kV. For each treatment, over ten fields were observed, and representative images were acquired.

**Metagenomic sequencing and analyses**

The total community DNA was extracted using a DNeasy® PowerWater® Kit (Qiagen, Hilden, Germany) according to the manufacturer’s instructions. The extracted DNA was dissolved in 100 μL Tris-HCl buffer and quantified based on OD (OD_260_/OD_230_ > 1.8) using a NanoDrop Lite spectrophotometer. DNA sequencing and analyses were conducted following steps described in our recent studies [37,38,39]. In detail, all 27 samples were subjected to metagenomic sequencing individually on the Illumina NovaSeq 6000 sequencing platform at the Novogene Bioinformatics Institute (Beijing, China). Paired-end reads with 150-bp read length were generated after the construction of libraries with 350-bp insertion. For each sample, more than 20 Gb of data were generated. Quality control of the Illumina sequences was performed on our local server using the software NGS QC Toolkit (version 2.3.3) [40]. We then removed reads containing adaptors, low-quality (quality score < 20) reads, or unpaired high-quality reads. Parallel-META3 [41] was used for taxonomic classification based on the miTags extracted from the metagenomes. PCoA based on Bray-Curtis and Jaccard methods was used to study the dissimilarity between the 27 samples by using the software PAST (v2.0) [42].

For functional analysis, the Illumina sequences were assembled by using MEGAHIT (version v1.2.x) [43] using the default kmer series. ORFs were predicted using Prodigal (version 2.6.3) [44] in the metagenome mode and only ORFs with a close end were retained. Then the ORFs were clustered using cd-hit (similarity cutoff > 90%) to generate orthologs with similarity. The orthologs were annotated by searching against the 2022 updated version of the KEGG database [45] using basic local alignment search tool (BLAST)-p (E-value < 1e−7). To calculate the relative abundance of predicted ORFs in the metagenome of each sample, all the metagenomes included for analyses were normalized to 2,000,000 reads. The metagenomic reads were mapped to all predicted ORFs using Diamond BLASTx (E-value < 1e−7 and similarity > 95%) [46]. Relative abundance was calculated by counting the number of mapped reads. The comparison of enriched KEGG genes among groups (0.3-MP-10, 3-MP-10, 3-GP-10, 0.3-GP-10, and seawater) were conducted using one-way ANOVA followed by false discovery rate (FDR) correction. The comparison of KEGGs between the 3-MP-10 and the 0.3-MP-10 biofilms were also conducted using DESeq2 [47], and the recruited metagenomic reads numbers were set as queries.

**16S rRNA gene amplicon sequencing and analyses**

For the Illumina sequencing, a 50-μL PCR amplification system was prepared for each sample (n = 27). The system consisted of 25 μL of Prime Star Max premix (R045, Takara, Beijing, China), 1 μL of DNA template, 2.5 μL of 16S-341F (5′-CCTACGGGNGGCWGCAG-3′) and 2.5 μL of 16S-805R primers (5′-GACTACHVGGGTATCTAATCC-3′) with different barcodes, and distilled water to the remaining volume. The PCR amplification program included a denaturation step at 95 °C for 5 min, 30 cycles of amplification (95 °C for 30 s, 57 °C for 30 s, and 72 °C for 2 min), and a final extension step at 72 °C for 5 min. The PCR products were examined using agarose gel electrophoresis and a Qubit assay. Libraries were generated using TruSeq® DNA PCR-Free Sample Preparation Kit (Illumina, USA) following the manufacturer’s protocol. Sequencing was conducted on an Illumina PE250 platform by Novogene (Beijing, China), with a depth of 50,000 pair-ended reads per sample.

After sequencing, paired-end reads were assigned to samples according to their unique barcodes. After cutting off the barcode and primer sequences, the paired-end reads were merged using FLASH [48]. For chimera removal, the sequences were compared with the Silva database using UCHIME algorithm [49]. Then Parallel-META3 was used to classify the Illumina clean reads using a similarity cutoff of 97%, and OTUs with less than two reads were removed. The mapped OTU IDs in the Parallel-META3 database file were used as the first column to construct an OTU table, in which OTUs with less than two reads (total number) were removed. The relative abundance of a given OTU was indicated by the read number it contained. Alpha-diversities based on the Chao1 and Shannon (H) indexes were calculated using the script alpha_diversity.py documented in the software package QIIME2 [50]. The scripts multiple_rarefactions.py, alpha_diversity.py, and collate_alpha.py script documented in QIIME2 were used to draw rarefaction curves at intervals of 100 (for Chao1) or 500 (for Shannon) with 10 replicated calculations. Taxonomic classification was performed based on the OTU table annotated by the database associated with Parallel-META3. The relative abundance of a given phylum or genus was represented by summarizing the read numbers in all the respective OTUs. PCoA was performed using PAST after transforming the OTU compositions into a Bray-Curtis distance and Jaccard matrix.

**Metatranscriptomic sequencing and analyses**

The 3-MP-10 and the 0.3-MP-10 biofilms were subjected to metatranscriptomic sequencing and analyses. For each group, three replicates were pooled for RNA extraction and sequencing, and thus, in total, two metatranscriptomes were sequenced. RNA extraction was performed using the TRIzol lysis method and Ribo-Zero Strand-specific libraries were prepared. Biotin-labeled oligonucleotides complementary to rRNA or other non-coding RNAs were mixed with the total RNA, and mRNA was selectively retained and converted to cDNA for library preparation. The libraries were sequenced on the HiSeq X Ten System in Novogene to generate 20 Gb data per metatranscriptome. Clean reads were obtained using the NGS QC Toolkit. The clean metatranscriptomic reads were mapped to all metagenome-derived orthologs using Diamond BLASTx (E-value < 1e−7 and similarity > 95%). The relative abundance was calculated by counting the number of mapped reads. For gene taxonomic affiliation analysis, the metatranscriptomic reads were assembled using MEGAHIT, and ORFs were predicted using Prodigal in a Meta and closed-end model. Taxonomic affiliation was determined by BLASTx searching (E-value < 1e−7) against the KEGG database (2022 version) and annotated by the genus_prokaryotes information file.

**qRT-PCR measurement**

Two sizes of MP were put in nylon mesh bags and immersed in the same subtidal zone of the DingjiaTsui to allow biofilm development for 5, 10, and 15 days, using an experimental period between February 2023 and March 2023. After transfer to the laboratory, the MPs were rinsed three times with sterile seawater to remove loosely attached cells. After ultrasonication and vortexing for 5 min, the bacteria attached to the particles were collected in sterile seawater and used for total RNA extraction and qRT-PCR measurement, conducted by the Tsingke Biotechnology Company (Beijing, China). The total RNA of bacterial cells was isolated using TRIzol reagent. After DNA digestion, an amount of 1.5 μg total RNA was reverse-transcribed to cDNA using the TIANScript II RT Kit (TIANGEN, Beijing, China) and the synthesized cDNAs served as templates in subsequent qRT-PCR reactions. Five pairs of primers (Table S4) were designed according to the five *flgE* genes assembled and predicted from the 0.3-MP-10 biofilm metatranscriptomic data. Bacterial 16S V3-V4 genes were used as the internal reference. qRT-PCR assays were run in triplicate with the iCycler iQ5 thermocycler (Bio-Rad, USA). The reaction volume contained (10 μL) 12.5 μg cDNA, 5 μL SsoFast™ EvaGreen® Supermix (Bio-Rad, USA), 400 nM of each primer, and nuclease-free water to a ﬁnal volume of 10 μL. Reactions were performed under the following conditions: 95 °C for 2 min, followed by 40 cycles of 95 °C for 15 seconds, 60°C for 15 s, and then 72 °C for 20 s. A melting curve was created to conﬁrm that a single product was generated by each reaction. A negative control (water) was included in each run. PCR products were verified by agarose gel electrophoresis and sequencing. The relative expression levels of five *flgE* genes were calculated relative to the expression of the internal control gene and the following formula was used to calculated the relative expression level of each *flgE*:

$$Y=2^{-\triangle\triangle Ct}$$

$$\triangle\triangle Ct=\triangle Ct-mean\triangle Ct$$

Where △Ct represents the differences in the cycle threshold value of the target *flgE* gene and the control gene products, mean △Ct represents the average △Ct value of the control group. Mean values were obtained from three biological replicates.

**Bacterial isolation and identification**

Bacteria were isolated from MPs deployed in the subtidal zone. After transport to the laboratory, bacterial cells were scraped off the MPs using sterile cotton tips and resuspended in the marine broth 2216E medium. The cells were then serially diluted (10, 10^2^, 10^3^, 10^4^, and 10^5^-fold dilutions), plated on 2216E agar plates, and incubated in an incubator at 25 ℃. Single colonies were picked up and purified for at least five times by passage culture. PCR of the 16S rRNA gene was conducted in a Prime Star Max premix (Takara, Beijing, China) with the primer pair 27F: 5′-AGAGTTTGATCCTGGCTCAG-3′ and 1492R: 5′-GGTTACCTTGTTACGACTTC-3′. Sanger sequencing of the PCR products was performed in Sangon Biotech (Shanghai, China) to obtain the full-length 16S rRNA genes. The 16S rRNA gene sequences were used as queries to BLASTn search against the NCBI Nucleotide database for taxonomic identification, using the default parameters.

**Genome sequencing and analyses**

Whole genomic DNA was extracted from the PMMA93 culture using a FastPure® Bacteria DNA Isolation Mini kit (Vazyme, Nanjing, China). The DNA was sequenced on the PacBio CCS and the Illumina NovaSeq platforms in Novogene (Tianjin, China). The complete genome was obtained by mix-assembly of the PacBio and Illumina reads using SPAdes (version 3.0.0) [51] installed on a local Linux system. The ORFs and corresponding protein sequences were predicted using Prodigal following the above-described criteria. Functional annotation of the genes was performed using Diamond BLASTp (E-value < 1e−7) by searching the protein sequences against the KEGG database (2022 version). The online software KEGG Mapper (https://www.genome.jp/kegg/mapper.html) was used to reconstruct the metabolic pathways based on the annotated proteins.

**Motility assay**

Motility assays were performed on two isolated strains. The marine broth 2216E medium (Sangon Biotech, Shanghai, China) was solidified with Noble agar (Sangon Biotech, Shanghai, China) in 0.3% and 0.5% concentrations. Bacterial cells grown in the 2216E liquid medium were harvested at the log growth phase (OD_600_ = 0.7), seeded on the agar plates (0.3 cm below the surface), and incubated under aerobic conditions. The seeded plates were incubated in an incubator at constant moisture (10%) and temperature (25 ℃) and the colony sizes were recorded. Each treatment was performed in triplicate.

**Bacterial colonization experiment and cell enumeration**

Bacterial cells grown to the log phase in the 2216E liquid medium were used for the colonization experiment. The cells were incubated with 3-MPs or 0.3-MPs and different concentrations of CCCP in 6-well plates (Sangon Biotech, Shanghai, China) at 25 ℃ for 12 h. At these concentrations, CCCP had no impact on the growth of the tested strain. Cells attached to the MPs were washed off using autoclaved 2216E medium and quantified at OD_600_ in a Biotek Cytation5 imaging reader (Biotek Instruments, Winooski, USA). Each treatment was performed in triplicate. Alternatively, cells attached to the MPs were washed off, diluted using 2216E medium and plated on 2216E agar plates. After incubation at 25 ℃ for 24 h, bacterial colonies were enumerated. Each treatment was performed in triplicate.

**Bacterial co-culture and colonization**

Two strains were grown to the log phase in the 2216E liquid medium and mixed in a 1:1 ratio. Bacterial cells were incubated with 3-MPs or 0.3-MPs in 6-well plates at 25 ℃ for 12 h. Bacterial cells on the MPs and in the media were collected, followed by DNA extraction and 16S rRNA gene amplification using the V3V4 universal primers (16S-341F and -805R as listed above). Libraries were constructed and then sequenced on an Illumina PE250 platform by Novogene (Beijing, China), with a depth of 30,000 pair-ended reads. Clean reads were mapped to 16S rRNA gene sequences predicted from the bacterial genomes using Bowtie2 [52]. The number of mapped reads was used to indicate bacterial percentages after normalization based on the 16S rRNA gene copy numbers of the two target strains. Each treatment and respective sequencing was performed in triplicate.

**Gene knockout**

Knockout of the *motA* in *V. alginolyticus* 2-8 was done following the method described in our previous studies [53,54,55]. In detail, the 790-bp upstream fragment and the 775-bp downstream fragment flanking *motAB* genes were amplified with primer pairs *motAB*-up-F/*motAB-*up-R and *motAB*-down-F/*motAB*-down-R (Table S7). The upstream and downstream fragments were ligated by overlap PCR. The resulting products were inserted into the SphⅠ/SalⅠ sites of the suicide vector pDM4 to yield the knock-out plasmid pDM4-Δ*motAB*, which was then transformed into *Escherichia coli* S17-1. After the conjugation between *E. coli* S17-1(pDM4-*ΔmotAB*) and *V. alginolyticus* 2-8 on TSBNa (supplemented with 3% NaCl) agar plates at 28 ℃ for 48 h, the cells were resuspended in PBS and plated onto TSBNa agar plates containing 30 μg ml-1 of chloramphenicol and 100 μg ml-1 of kanamycin, which selected *V. alginolyticus* transconjugants. About 20 candidate colonies were transferred to TSBNa medium and incubated at 28 ℃ overnight before spread on TSBNa agar plates containing 12% sucrose. Double cross-over mutants resulting in the nonpolar knock-out of *motAB* were verified by PCR using primer pairs *motAB*-up-F/*motAB*-down-R and Sanger DNA sequencing.

**Statistical analyses**

Two-tailed Students’ t-tests were performed in MS Excel, after using the Levene's test to confirm the equality of variances. For multiple *p*-values, adjustment based on the FDR rate was performed using the “fdrtool” function in R (v 4.2.1) [56]. One-way ANOVA was performed in R through the “car” and “aov” functions. The *p*-values were adjusted based on the FDR rate in the “fdrtool” function. DESeq2 was also performed in the R software through the “DESeq2” package, and the *p*-values were adjusted by FDR associated with DESeq2.

**REFERENCES**

1. Lu, Jie, Yi Shu, Heng Zhang, Shangxian Zhang, Chengrui Zhu, Wei Ding, and Weipeng Zhang. 2023. “The Landscape of Global Ocean Microbiome: From Bacterioplankton to Biofilms.” *International Journal of Molecular Sciences* 24: 6491. https://doi.org/10.3390/ijms24076491
2. Zhang, Weipeng, Wei Ding, Yong-Xin Li, Chunkit Tam, Salim Bougouffa, Ruojun Wang, Bite Pei, et al. 2019. Marine Biofilms Constitute a Bank of Hidden Microbial Diversity and Functional Potential. *Nature Communications* 10:517. <https://doi.org/10.1038/s41467-019-08463-z>
3. Ding, Wei, Ruojun Wang, Zhicong Liang, Rui Zhang, Pei-Yuan Qian, and Weipeng Zhang. 2021. “Expanding Our Understanding of Marine Viral Diversity through Metagenomic Analyses of Biofilms.” *Marine Life Science & Technology* 3: 395-404. https://doi.org/10.1007/s42995-020-00078-4
4. Ding, Wei, Shougang Wang, Peng Qin, Shen Fan, Xiaoyan Su, Peiyan Cai, Jie Lu, et al. 2023. “Anaerobic Thiosulfate Oxidation by the *Roseobacter* Group Is Prevalent in Marine Biofilms.”*Nature Communications* 14: 2033. https://doi.org/10.1038/s41467-023-37759-4
5. Patel, Ravi K., and Mukesh Jain. 2012. “NGS QC Toolkit: A Toolkit for Quality Control of Next Generation Sequencing Data.” *PLoS One* 7: e30619. <https://doi.org/10.1371/journal.pone.0030619>
6. Jing, Gongchao, Zheng Sun, Honglei Wang, Yanhai Gong, Shi Huang, Kang Ning, Jian Xu, and Xiaoquan Su. 2017. “Parallel-META 3: Comprehensive Taxonomical and Functional Analysis Platform for Efficient Comparison of Microbial Communities.” *Scientific Reports* 7: 40371. <https://doi.org/10.1038/srep40371>
7. Hammer, Øyvind, David A. T. Harper, and Paul D. Ryan. 2001. “Past: Paleontological Statistics Software Package for Educaton and Data Analysis.” *Palaeontologia Electronica* 4: 4-9. https://doi.org/ 10.1016/j.palaeo.2009.11.002
8. Li, Dinghua, Chi-Man Liu, Ruibang Luo, Kunihiko Sadakane, and Tak-Wah Lam. 2015. “MEGAHIT: An Ultra-Fast Single-Node Solution for Large and Complex Metagenomics Assembly via Succinct de Bruijn Graph.” *Bioinformatics* 31: 1674-1676. https://doi.org/10.1093/bioinformatics/btv033
9. Hyatt, Doug, Gwo-Liang Chen, Philip F. Locascio, Miriam L. Land, Frank W. Larimer, and Loren J. Hauser. 2010. “Prodigal: Prokaryotic Gene Recognition and Translation Initiation Site Identification.” *BMC Bioinformatics* 11: 119. <https://doi.org/10.1186/1471-2105-11-119>
10. Kanehisa, [Minoru](https://pubmed.ncbi.nlm.nih.gov/?term=Kanehisa+M&cauthor_id=27899662), Miho Furumichi, Mao Tanabe, Yoko Sato, and Kanae Morishima. 2017. “KEGG: New Perspectives on Genomes, Pathways, Diseases and Drugs.” *Nucleic Acids Research* 45: 353-361. <https://doi.org/10.1093/nar/gkw1092>
11. Persson, Emma, and Erik L. L. Sonnhammer. 2022. “InParanoid-DIAMOND: Faster Orthology Analysis with the InParanoid Algorithm.” *Bioinformatics* 38: 2918-2919. <https://doi.org/10.1093/bioinformatics/btac194>
12. Love, Michael I., Wolfgang Huber, and Simon Anders. 2014. “Moderated Estimation of Fold Change and Dispersion for RNA-Seq Data with DESeq2.” *Genome Biology* 15: 550. <https://doi.org/10.1186/s13059-014-0550-8>
13. Magoč, Tanja, and Steven L. Salzberg. 2011. “FLASH: Fast Length Adjustment of Short Reads to Improve Genome Assemblies.” *Bioinformatics* 27: 2957-2963. https://doi.org/10.1093/bioinformatics/btr507
14. Edgar, Robert C., Brian J. Haas, Jose C. Clemente, Christopher Quince, and Rob Knight. 2011. “UCHIME Improves Sensitivity and Speed of Chimera Detection.” *Bioinformatics* 27: 2194-2200. https://doi.org/10.1093/bioinformatics/btr381
15. Bolyen, Evan, Jai R. Rideout, Matthew R. Dillon, Nicholas A. Bokulich, Christian C. Abnet, Gabriel A. Al-Ghalith, Harriet Alexander, et al. 2019. “Reproducible, Interactive, Scalable and Extensible Microbiome Data Science Using QIIME 2”. *Nature Biotechnology* 37: 852-857. https://doi.org/10.1038/s41587-019-0209-9
16. Bankevich, Anton, Sergey Nurk, Dmitry Antipov, Alexey A. Gurevich, Mikhail Dvorkin, Alexander S. Kulikov, Valery M. Lesin, et al. 2012. “SPAdes: A New Genome Assembly Algorithm and Its Applications to Single-Cell Sequencing.” *Journal of Computational Biology* 19: 455-477. <https://doi.org/10.1089/cmb.2012.0021>
17. Langmead, Ben, and Steven L. Salzberg. 2012. “Fast Gapped-Read Alignment with Bowtie 2.” *Nature Methods* 9: 357-359. <https://doi.org/10.1038/nmeth.1923>
18. Zhang, Weipeng, Yao Wang, Yunhong Song, Tietao Wang, Shengjuan Xu, Zhong Peng, Xiaoli Lin, Lei Zhang, and Xihui Shen. 2013. “A Type VI Secretion System Regulated by OmpR in *Yersinia* *pseudotuberculosis* Functions to Maintain Intracellular pH Homeostasis.” *Environmental Microbiology* 15: 557-569. https://doi.org/10.1111/1462-2920.12005
19. Lin, Jinshui, Weipeng Zhang, Juanli Cheng, Xu Yang, Kaixiang Zhu, Yao Wang, Gehong Wei, Pei-Yuan Qian, Zhao-Qing Luo, and Xihui Shen. 2017. “A *Pseudomonas* T6SS Effector Recruits PQS-Containing Outer Membrane Vesicles for Iron Acquisition.” *Nature Communications* 8: 14888. https://doi.org/10.1038/ncomms14888
20. Lin, Jinshui, Jianshe Yang, Juanli Cheng, Weipeng Zhang, Xu Yang, Wei Ding, Heng Zhang, Yao Wang, and Xihui Shen. 2023. “*Pseudomonas aeruginosa* H3-T6SS Combats H_2_O_2_ Stress by Diminishing the Amount of Intracellular Unincorporated Iron in a Dps-Dependent Manner and Inhibiting the Synthesis of PQS.” *International Journal of Molecular Sciences* 24: 1614. https://doi.org/10.3390/ijms24021614
21. Rizzo, Maria L. 2009. “Software for Data Analysis: Programming with R.” *The American Statistician* 63: 289.

**SUPPLEMENTARY FIGURES**


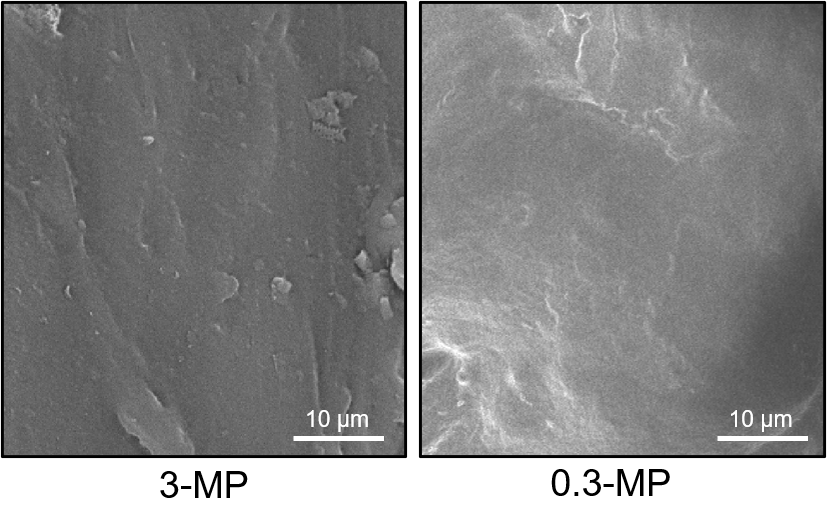


**FIGURE S1** Surface characters of the microplastic materials as observed by scanning electron microscope.

**
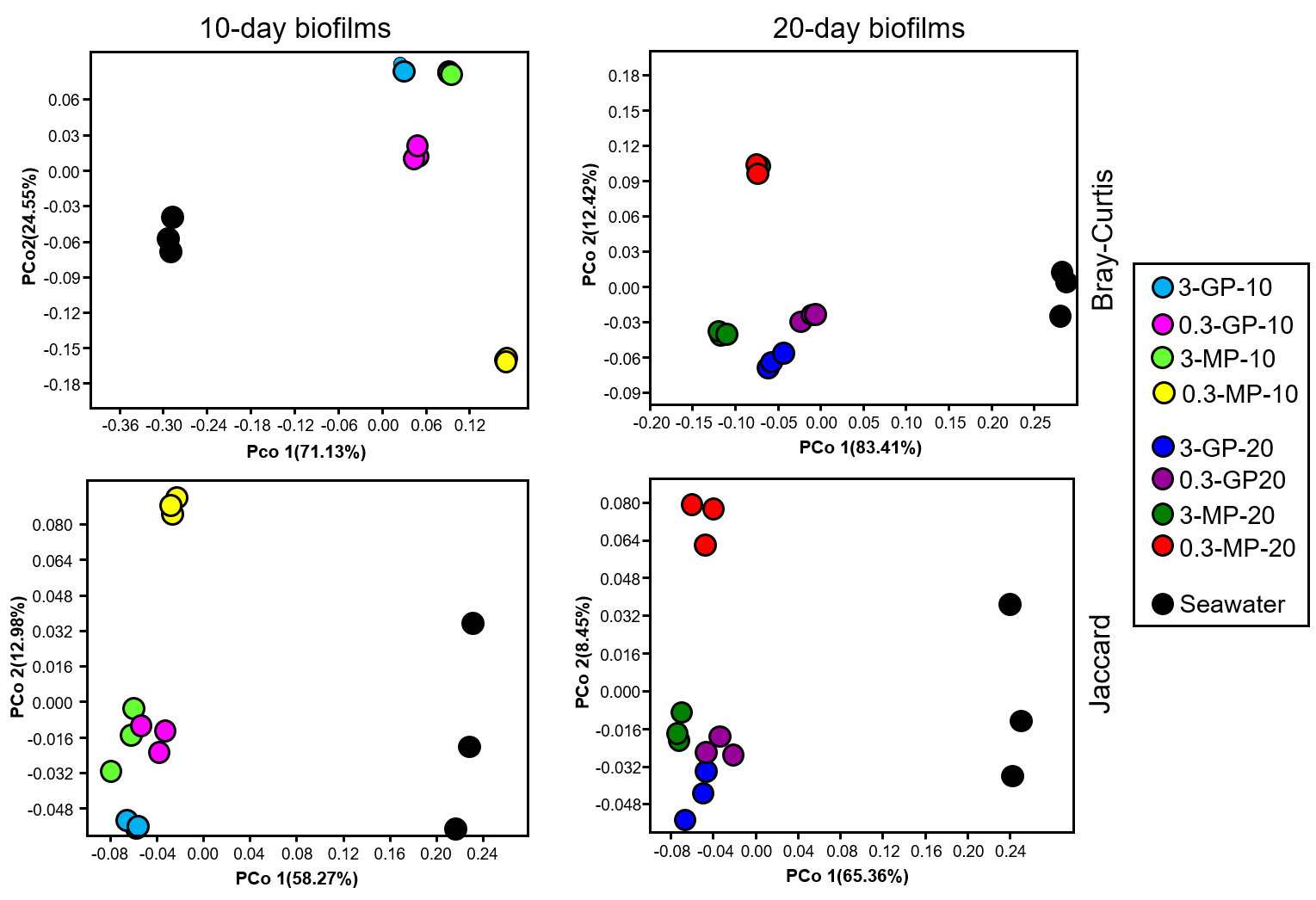
**

**FIGURE S2** Principal coordinate analysis of the 10-day biofilms, 20-day biofilms and seawater microbiota, based on the miTags. Bray-Curtis and Jaccard dissimilarities of the genus composition were observed.


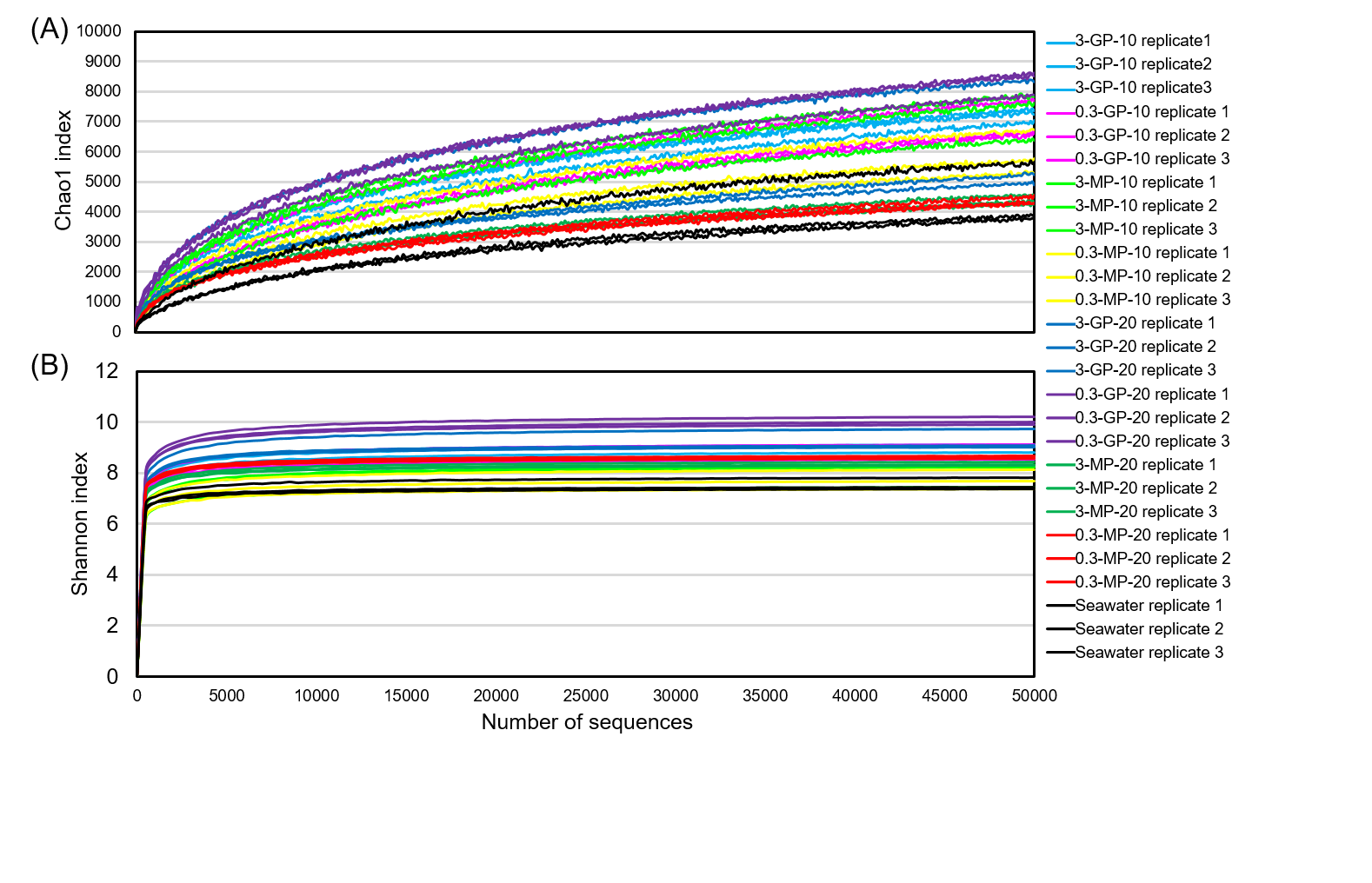


**FIGURE S3** Rarefaction curves of the 27 samples subjected to the 16S rRNA gene amplicon sequencing. The Chao1 (A) and Shannon index (B) between sequence numbers were calculated. The sequences were extracted with a stepwise of 100 (for Chao1) or 500 (for Shannon) and a permutation number of 10.


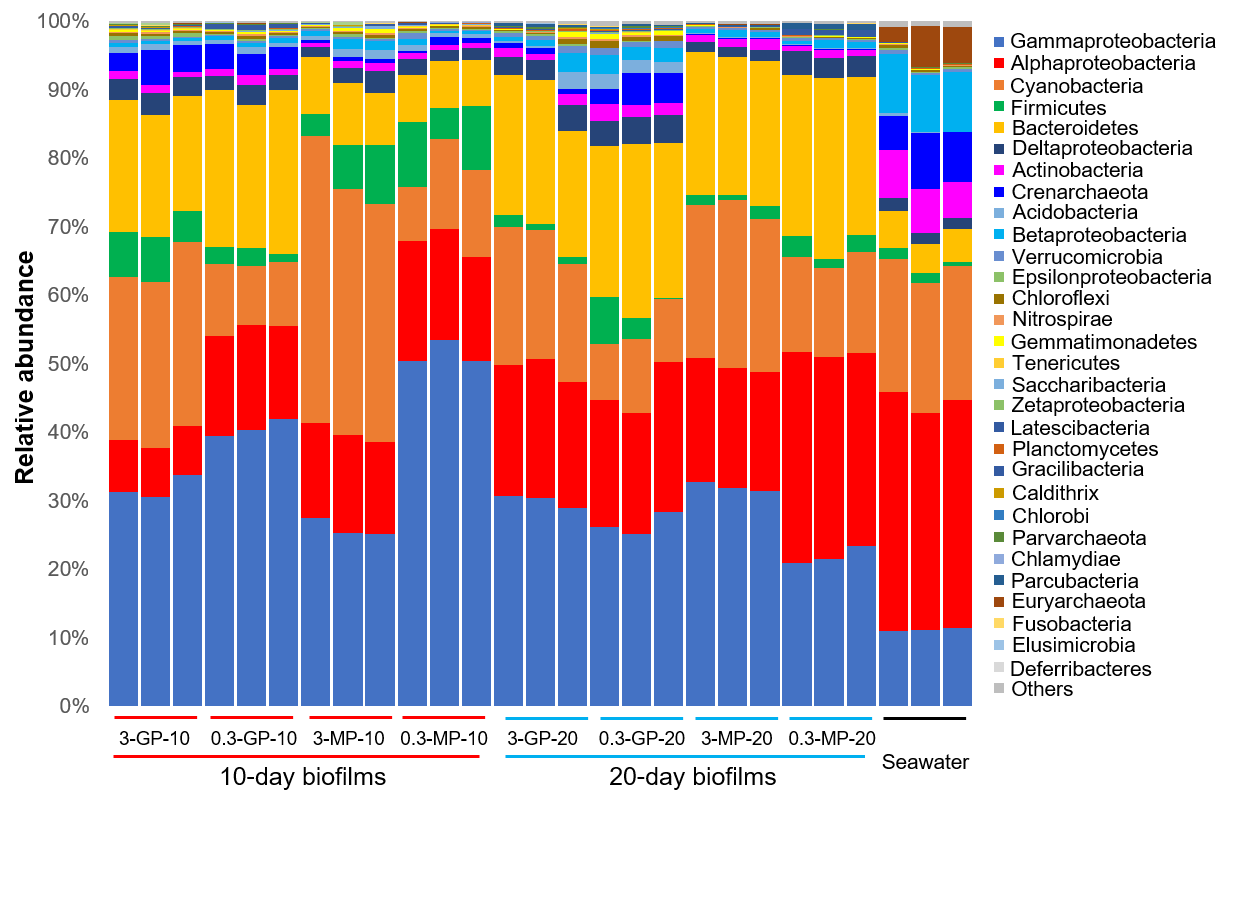


**FIGURE S4** Taxonomic composition of different samples at the phylum level based on the 16S rRNA gene amplicon sequences. Proteobacteria were classified down to the class level. The 30 most abundant phyla in terms of maximum relative abundance are listed, while the remaining was merged as “Others”.


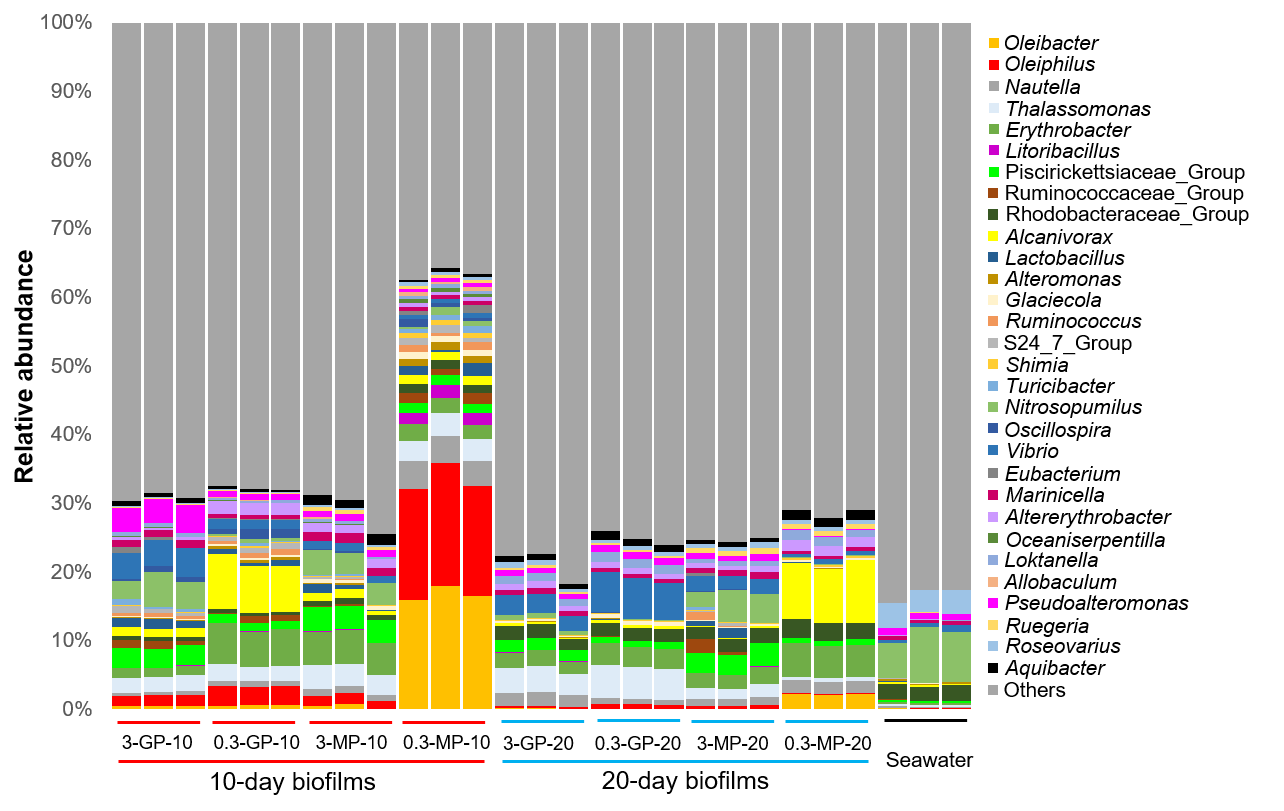


**FIGURE S5** Taxonomic composition of different samples at the genus level based on 16S rRNA gene amplicon sequences. The 30 most abundant phyla in terms of maximum relative abundance are listed, while the remaining was merged as “Others”.


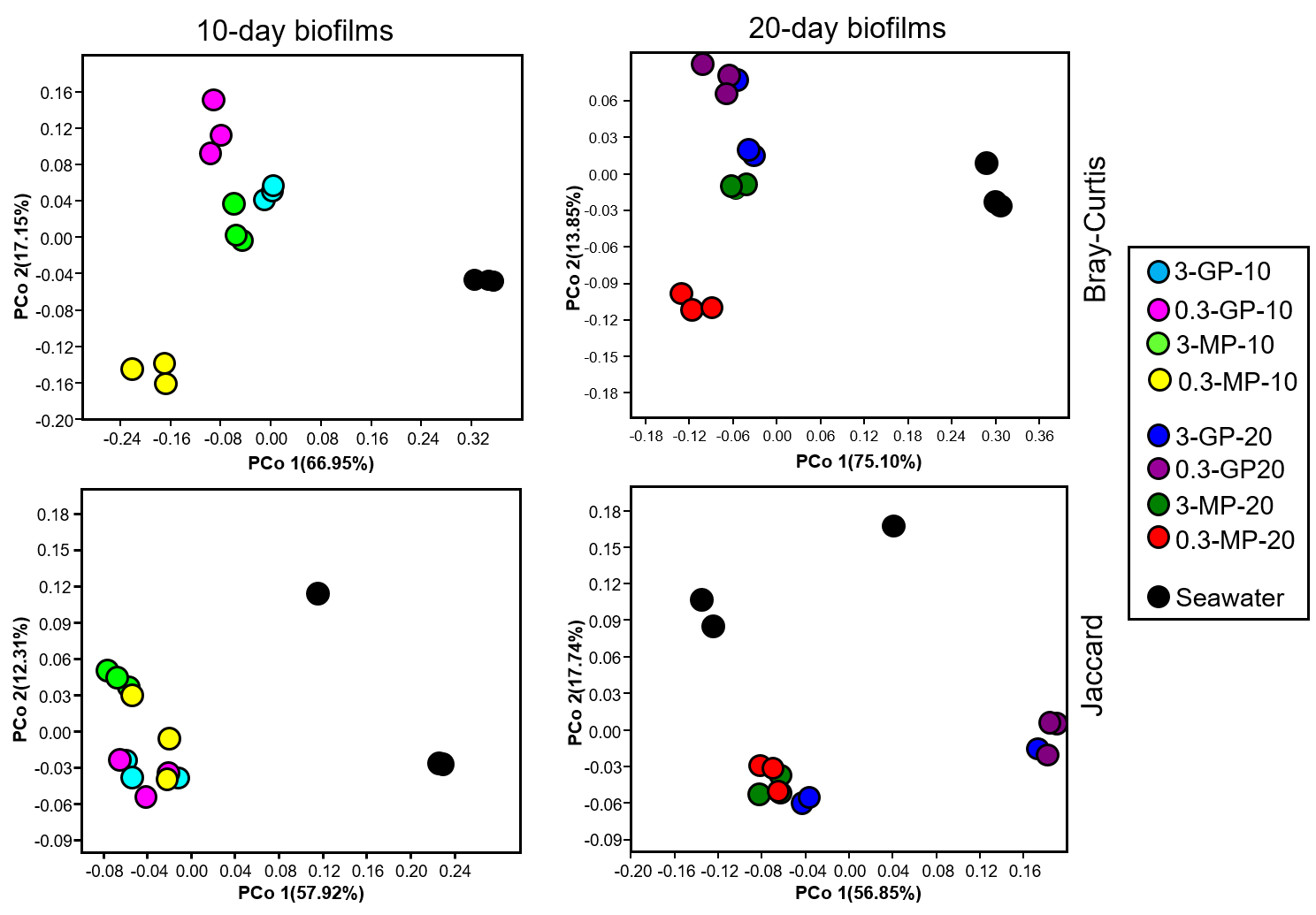


**FIGURE S6** Principal coordinate analysis of the genus-level structure of the 10-day biofilms, 20-day biofilms, and seawater microbiota, based on 16S rRNA gene amplicon sequences. Each group has three biological replicates. Bray-Curtis and Jaccard dissimilarities were calculated and visualized.


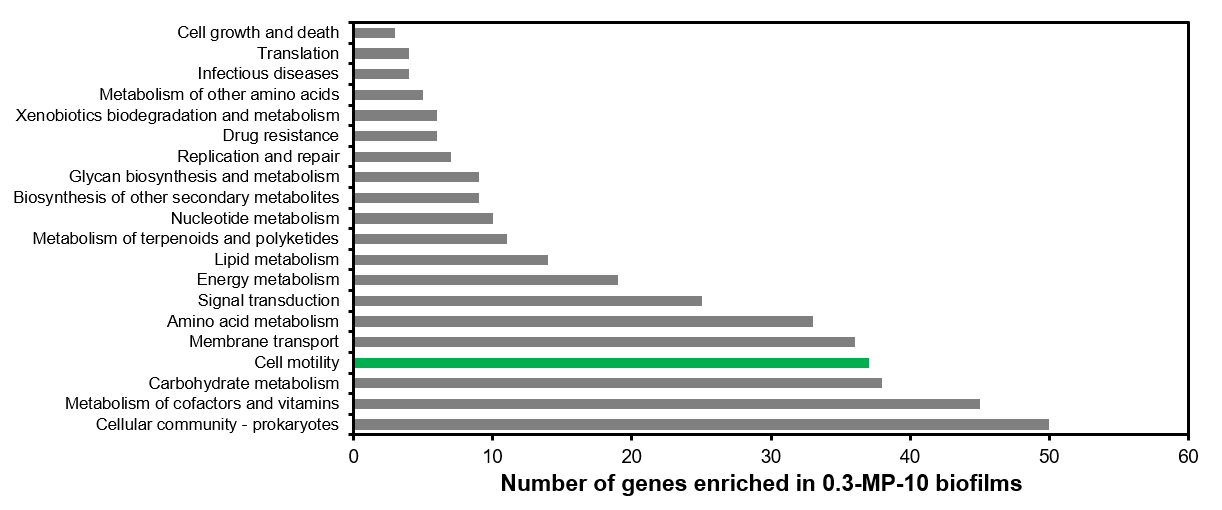


**FIGURE S7** Categories of the significantly enriched genes in the 0.3-MP-10 biofilms as revealed by metagenomic analyses. The genes were annotated by the Kyoto Encyclopedia of Genes and Genomes (KEGG) database and those in the 0.3-MP-10 biofilms were compared with those in the other four microbiota (3-MP-10 biofilms, 0.3-MP-10 biofilms, 3-GP-10 biofilms, 0.3-GP-10 biofilms, and seawater) to identify significantly enriched genes (adjusted *p*-value < 0.001 in one-way ANOVA).


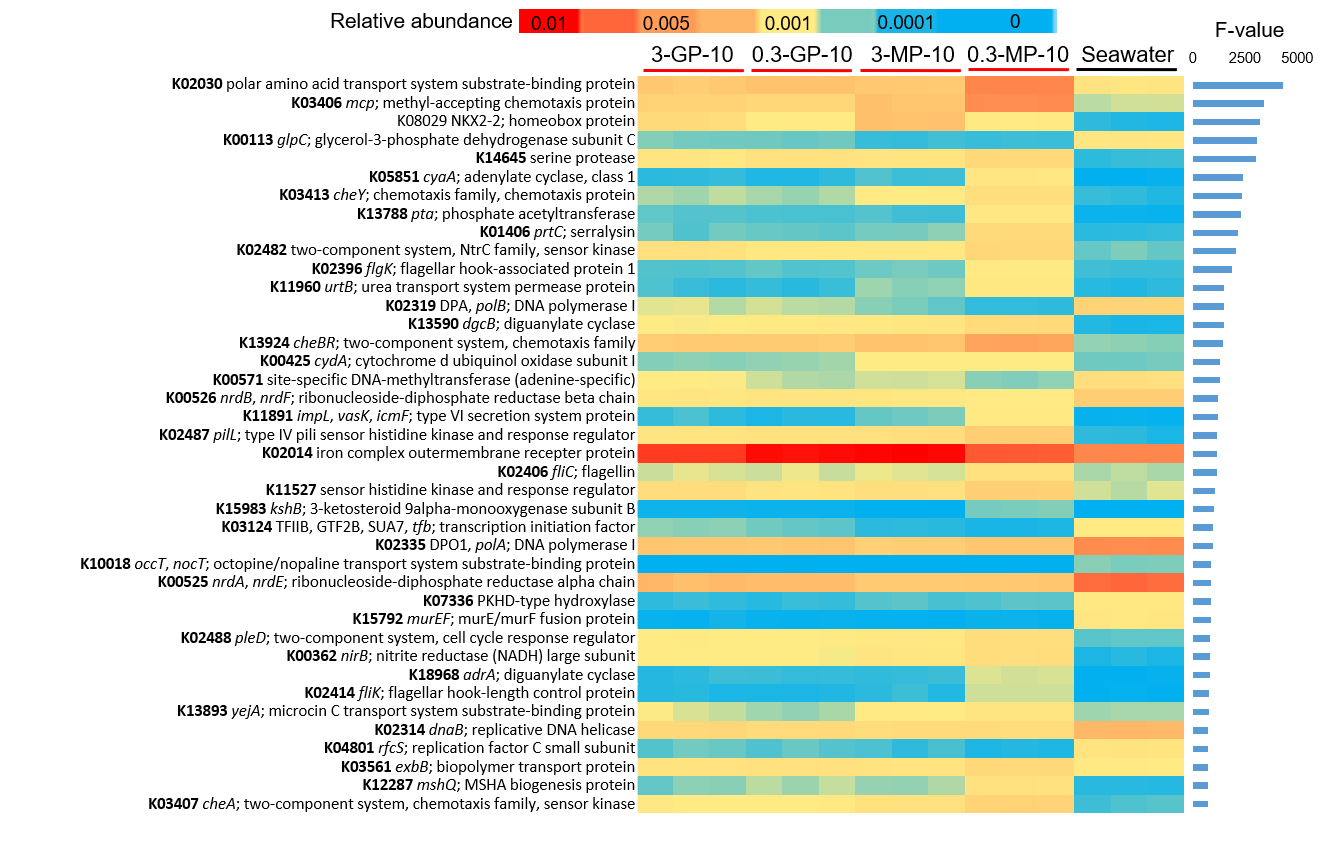


**FIGURE S8** Annotation and abundance distribution of the top 40 (indicated by F-value in one-way ANOVA) significantly differentially presented genes in the 10-day biofilms as revealed by metagenomic analyses. The genes were annotated by the KEGG database and those in the 0.3-MP-10 biofilms were compared with those in the other four microbiota (3-MP-10 biofilms, 0.3-MP-10 biofilms, 3-GP-10 biofilms, 0.3-GP-10 biofilms, and seawater) to identify significantly enriched genes (adjusted *p*-value < 0.001).

**
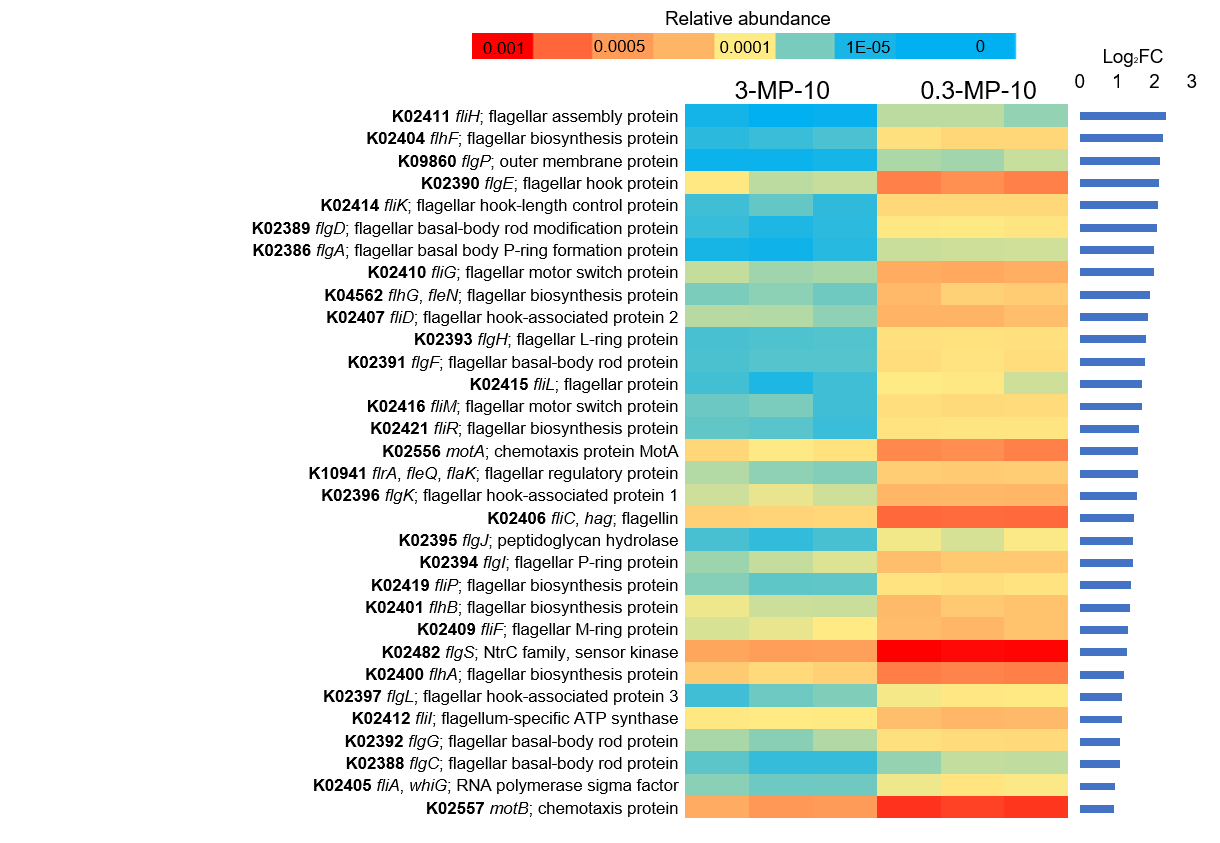
**

**FIGURE S9** The enrichment of flagellar-related genes in 0.3-MP-10 biofilms as revealed by DESeq2 analysis. Recruited reads number from the normalized metagenomes were used as queries during the DESeq2 test. It revealed 1,460 significantly-changed (adjusted *p*-value < 0.001) KEGG-annotated genes, and the relative abundance (reads percentage among the total reads recruited by all the genes) of flagellar-related genes are shown here. FC, fold change between 0.3-MP-10 biofilms and 3-MP-10 biofilms, as calculated by DESeq2.

**
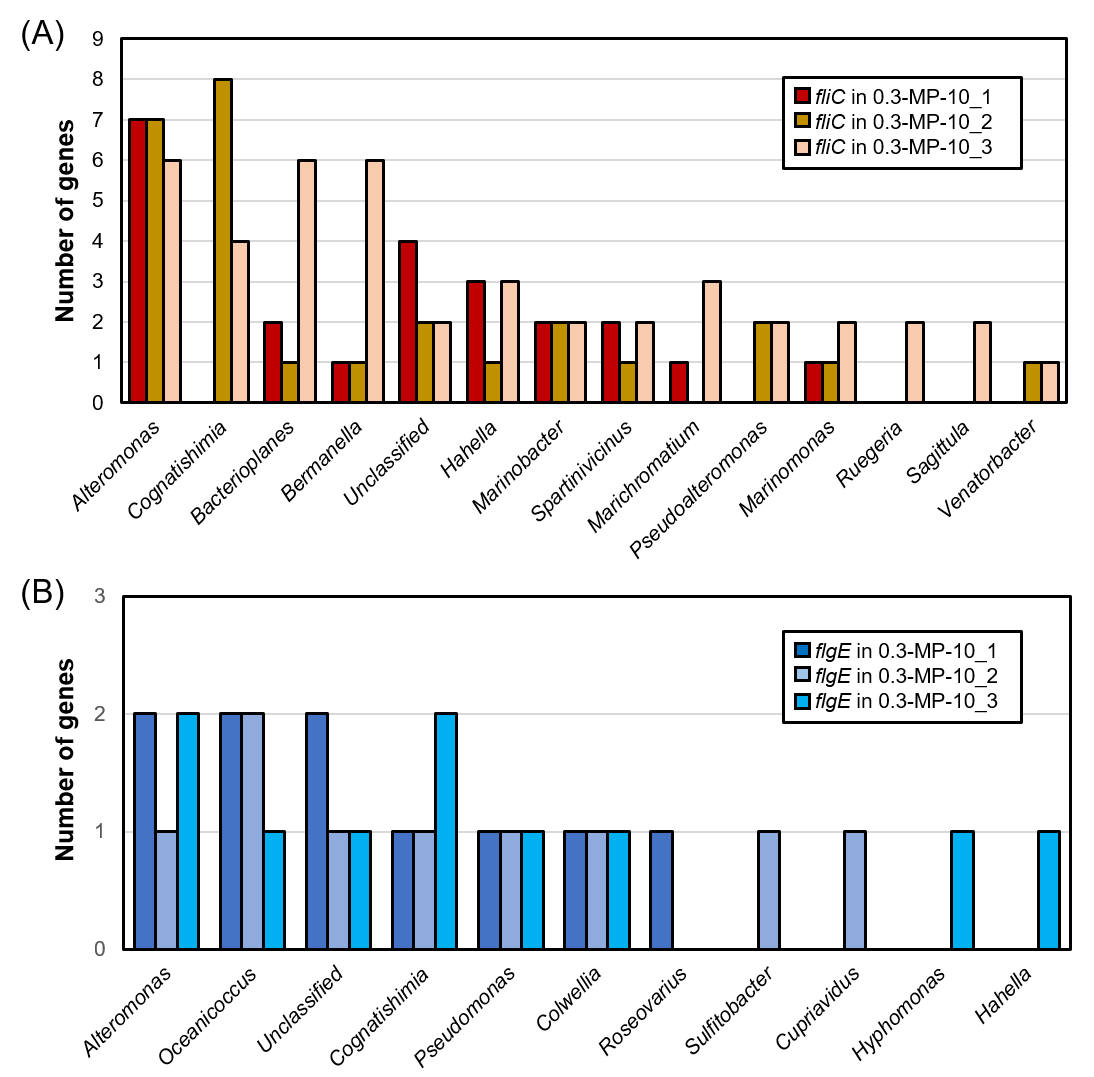
**

**FIGURE S10** Taxonomic affiliation (genus level) of the *fliC* (A) and *flgE* genes (B) in the assembled 0.3-MP-10 biofilm metagenomes. Taxonomic affiliation was determined by searching against the KEGG database and annotated by the “genus_prokaryotes” information file.

**
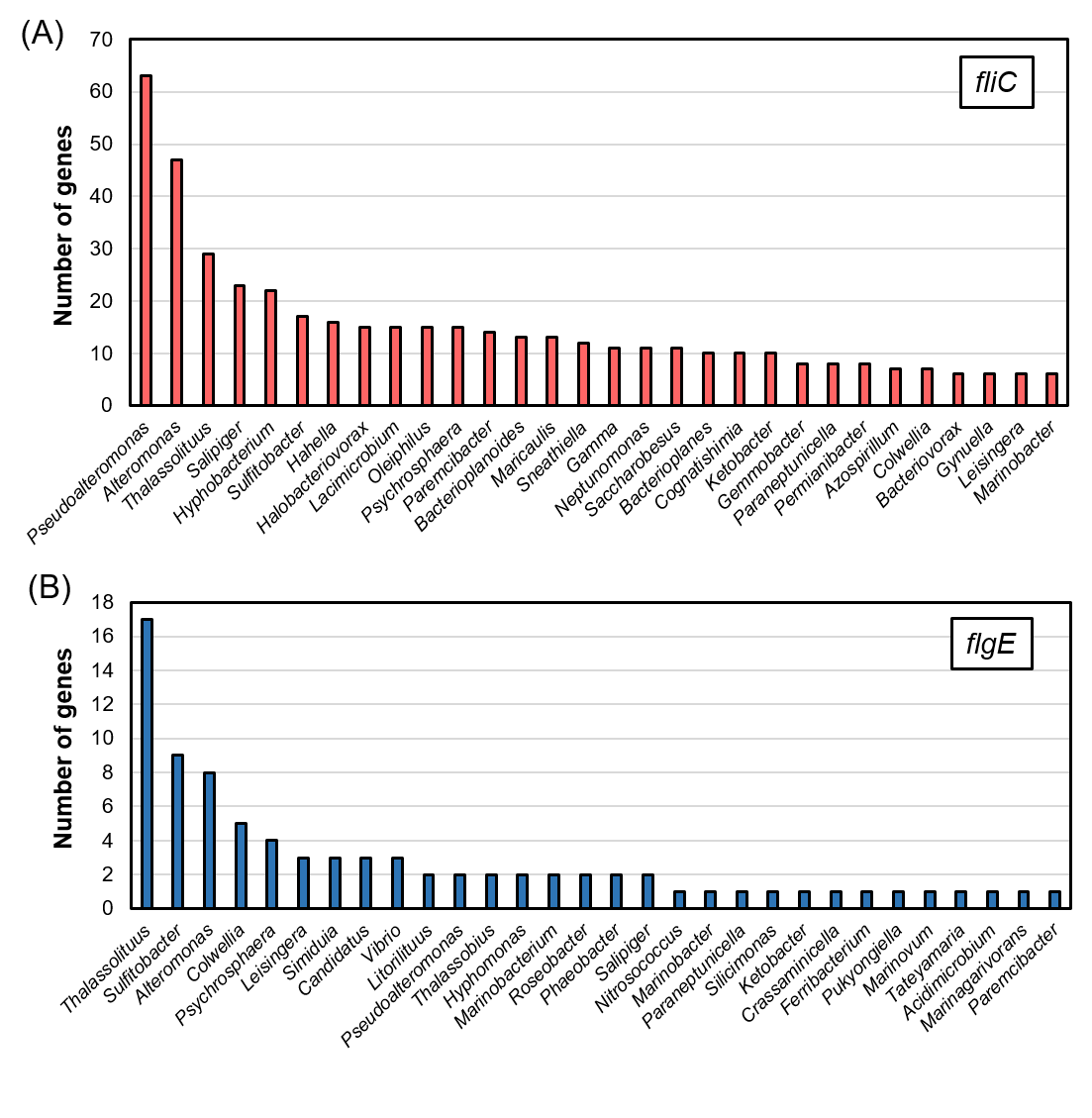
**

**FIGURE S11** Taxonomic affiliation (genus level) of the *fliC* (A) and *flgE* genes (B) in the assembled 0.3-MP-10 biofilm metatranscriptome. Taxonomic affiliation was determined by searching against the KEGG database and annotated by the “genus_prokaryotes” information file.


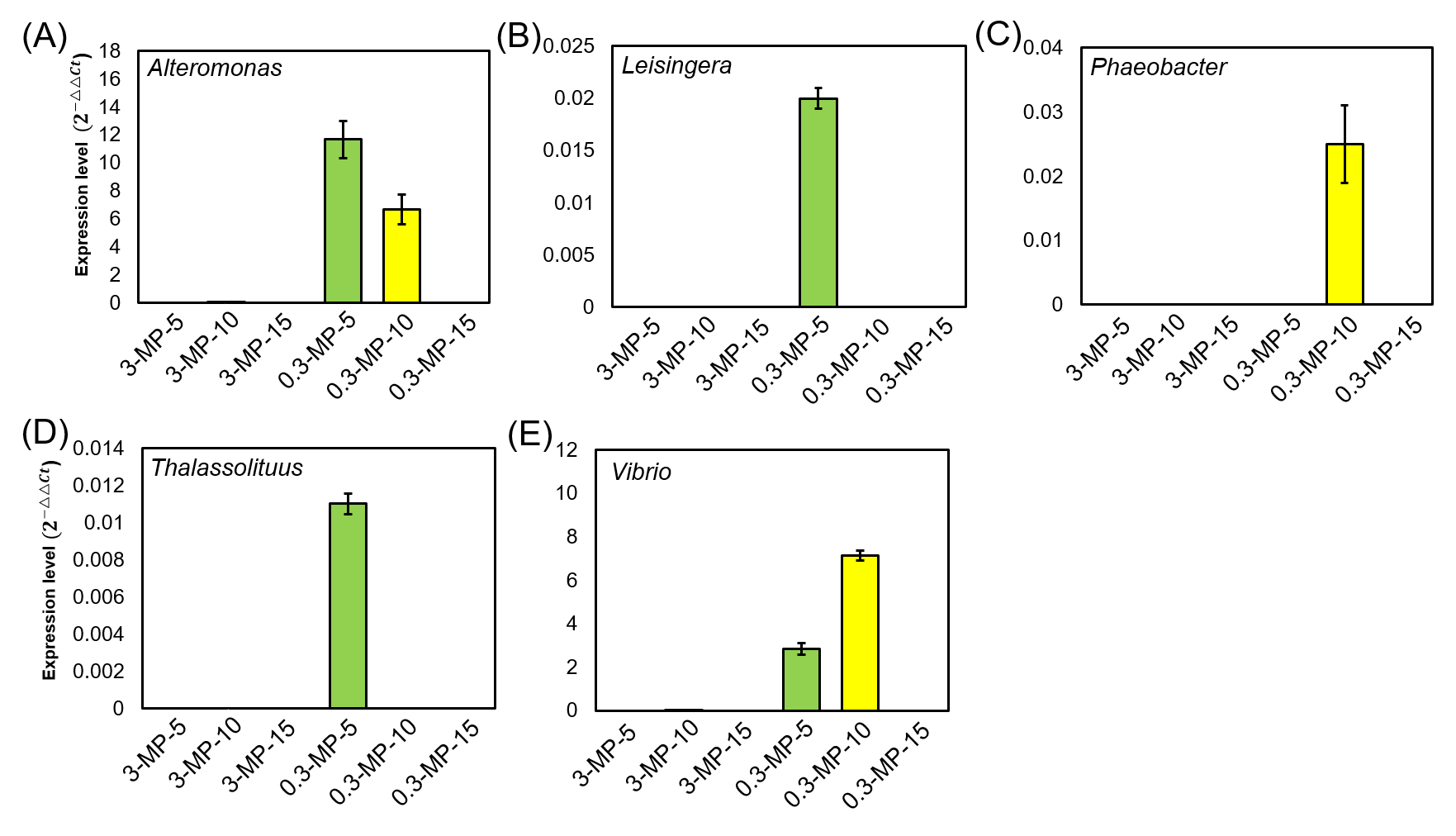


**FIGURE S12** qRT-PCR assay of the *flgE* genes in three stages of biofilms on larger or smaller MPs. The relative expression levels of five *flgE* genes from *Alteromonas* (A), *Leisingera* (B), *Phaeobacter* (C), *Thalassolituus* (D), and *Vibrio* (E) were indicated as percentage to the expression of the 16S rRNA genes. The error bar represents the standard deviation of three biological replicates. Students’ t-test was used to examine significant differences for the expressed genes, and *** represents *p*-value < 0.001.

**
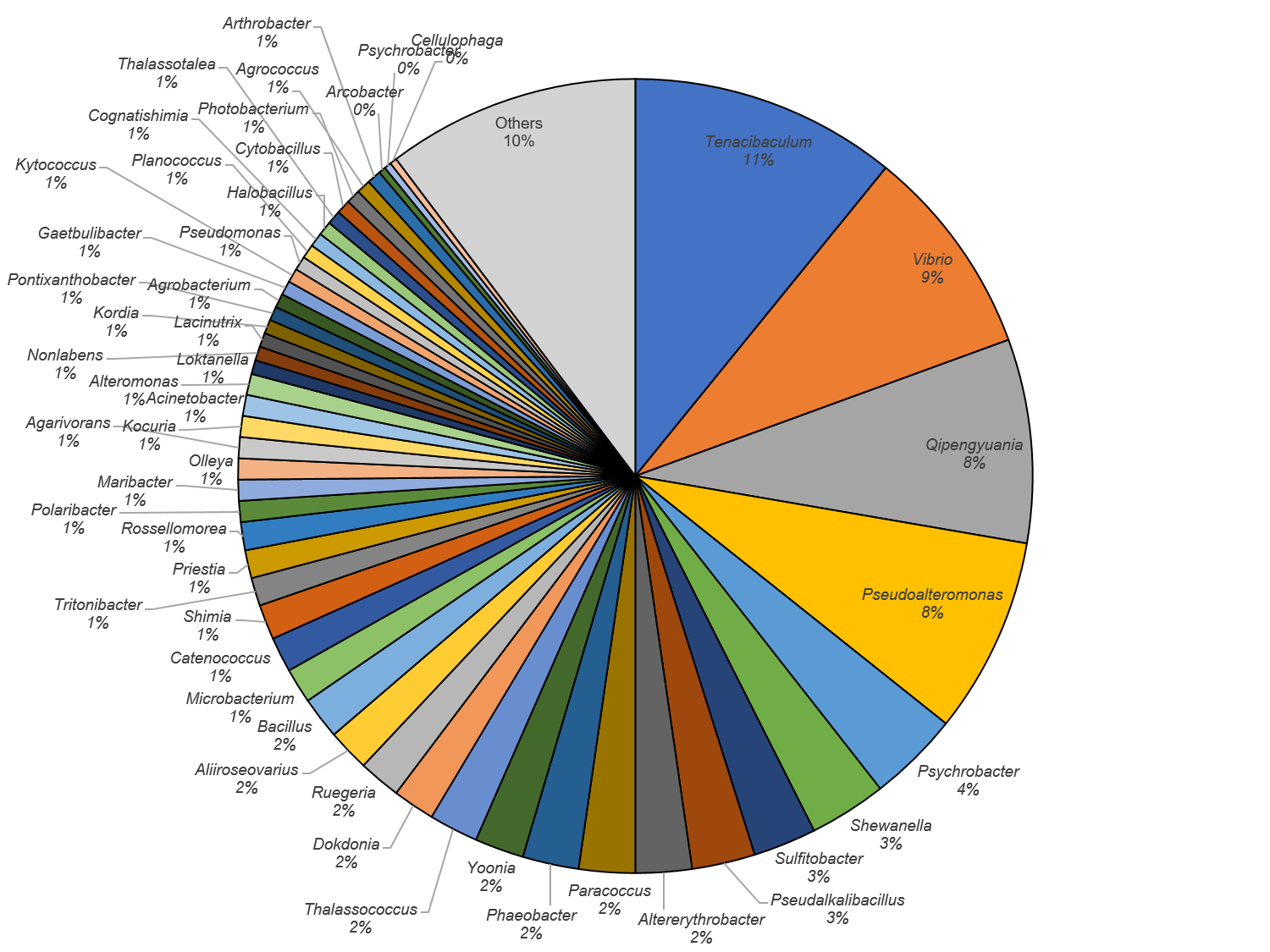
**

**FIGURE S13** Genus-level classification of all the strains isolated from marine MPs. This analysis was based on 16S rRNA genes generated by using Sanger sequencing.


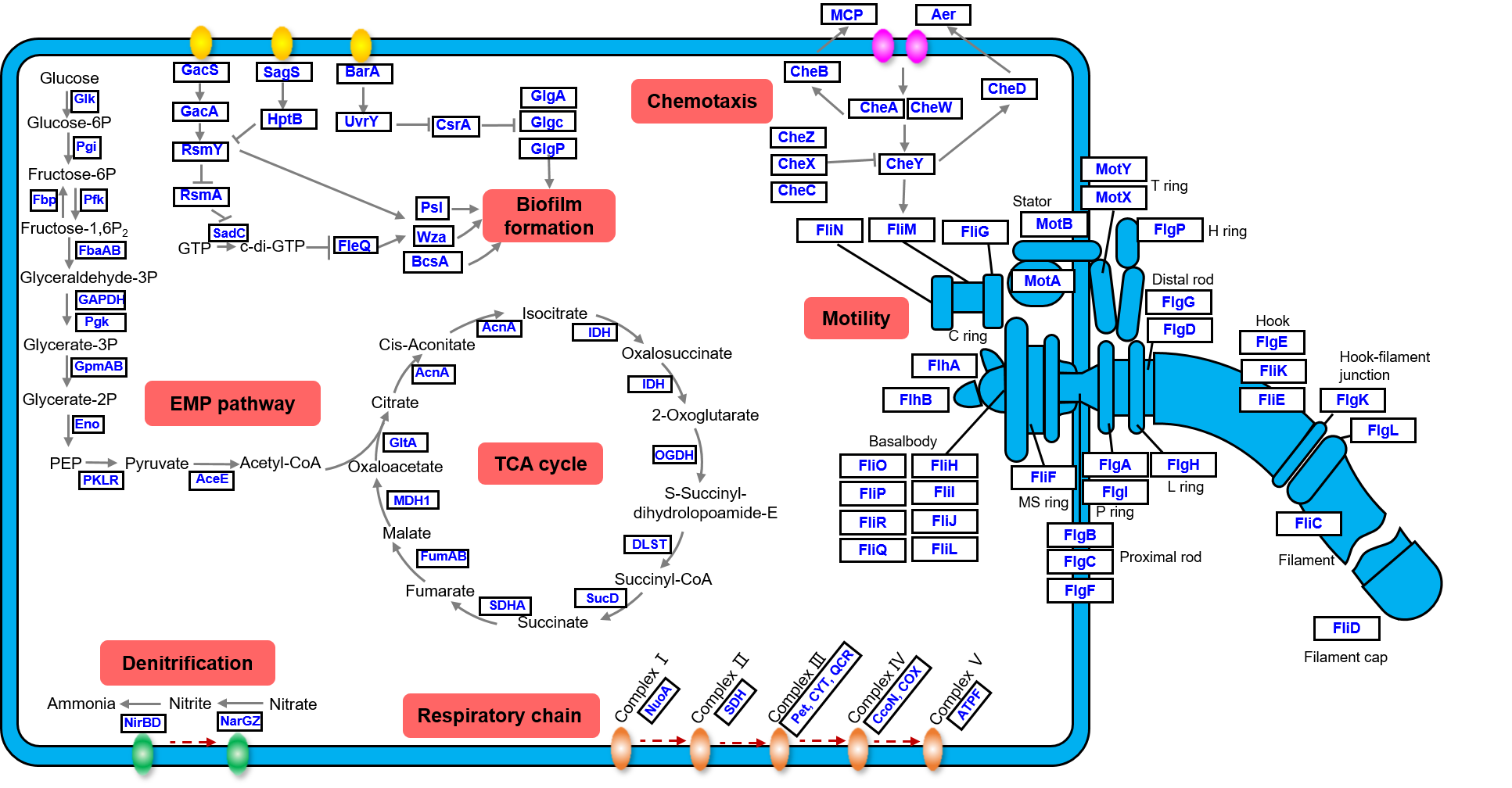


**FIGURE S14** Metabolic pathways of *Alteromonas* sp009811495 PMMA93. PMMA93 has a rather complete flagellar biosynthesis pathway, complete Embden-Meyerhof-Parnas (EMP) and tricarboxylic acid (TCA) pathways, and a number of genes responsible for biofilm formation and chemotaxis.

**
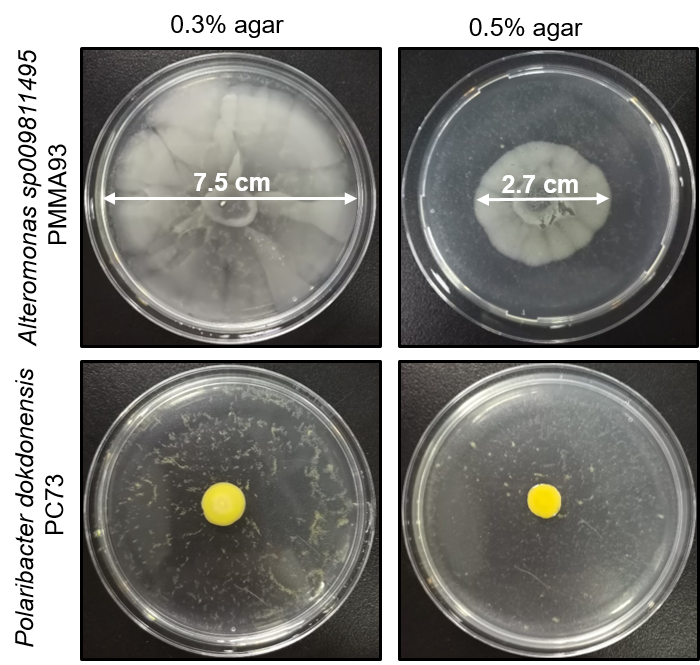
**

**FIGURE S15** Plate motility assay of *A.* sp009811495 PMMA93 under two agar concentrations. *Polaribacter dokdonensis* PC73, which was not detected in the microplastic particles metagenomes, was used as a negative control. The plates were placed at 25 ℃ for five days before observation.


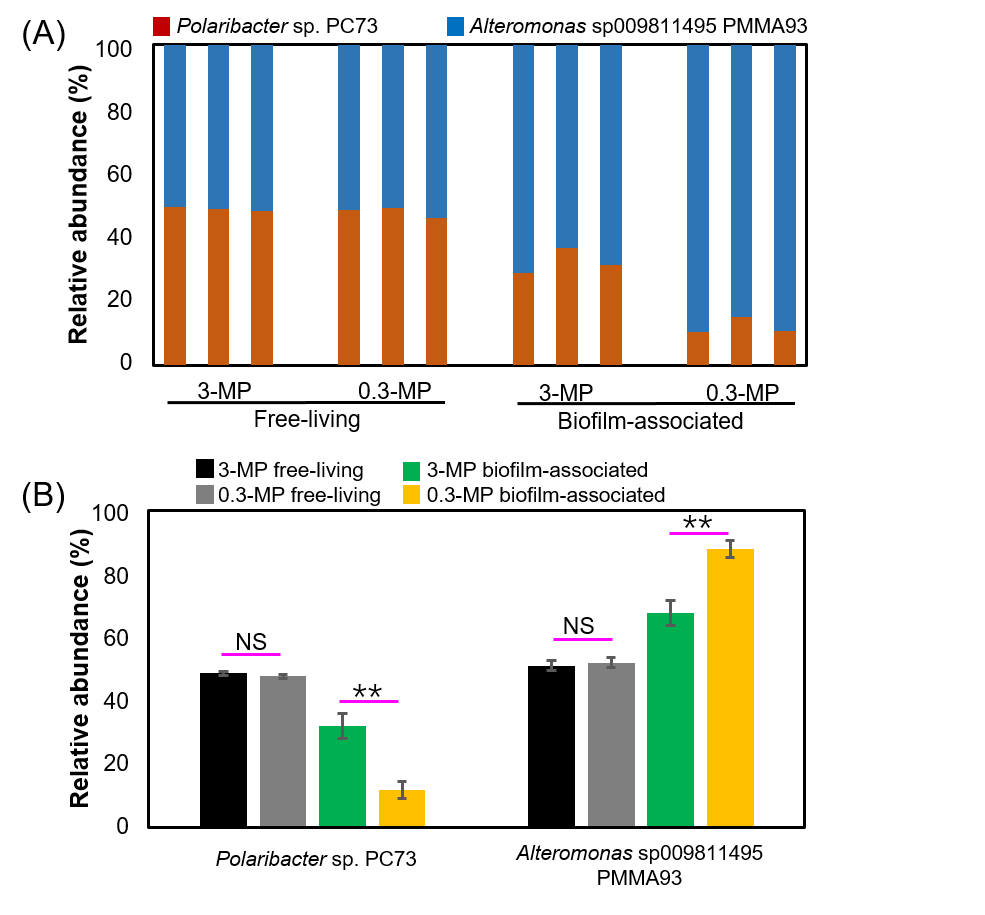


**FIGURE S16** Relative abundance of *P. dokdonensis* PC73 and *A.* sp009811495 PMMA93 after co-cultured in media with 3-MP or 0.3-MP. Bacterial cell abundances (indicated by 16S rRNA gene reads as revealed by amplicon sequencing) on the surface of microplastic particles (biofilm-associated) and in media (free-living) were analyzed. Individual results of the three biological replicates are shown in (A) and statistical results are shown in (B). ** represents *p*-value < 0.01 while NS indicates non-significance in Student’s t-test.


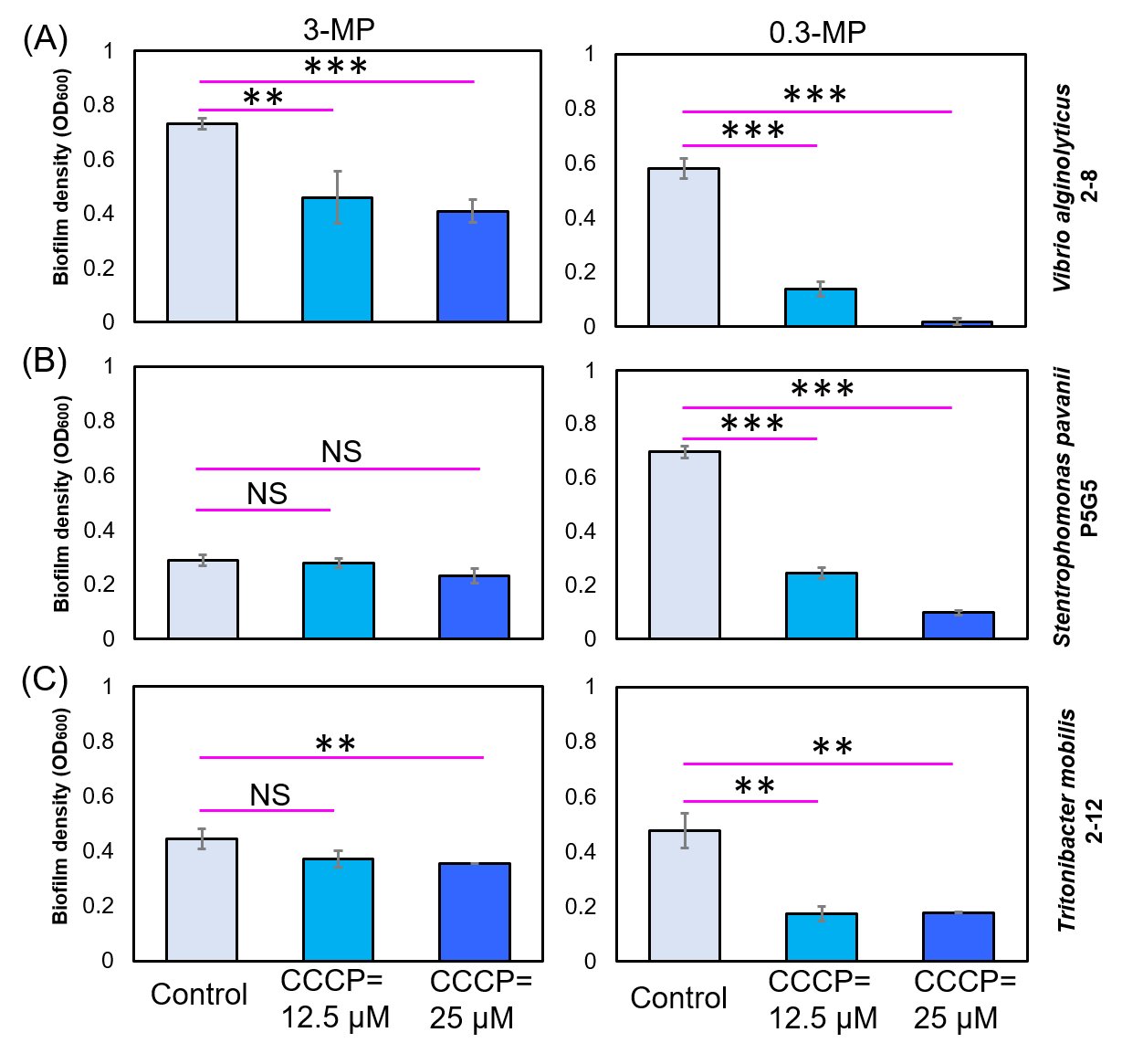


**FIGURE S17** The impact of CCCP on colonization of three bacterial strains on larger and smaller MPs. *Vibrio alginolyticus* 2-8 (A), *Stentrophomonas pavanii* P5G5 (B), and *Tritonibacter mobilis* 2-12 (C) were isolated from marine MPs, and incubated with the 3-mm MPs or 3-mm MPs along with the addition of 0, 12.5, and 25 μm CCCP for 12 h, followed by cell density measurement. ** represents *p*-value < 0.01 while NS indicates non-significance in Student’s t-test.


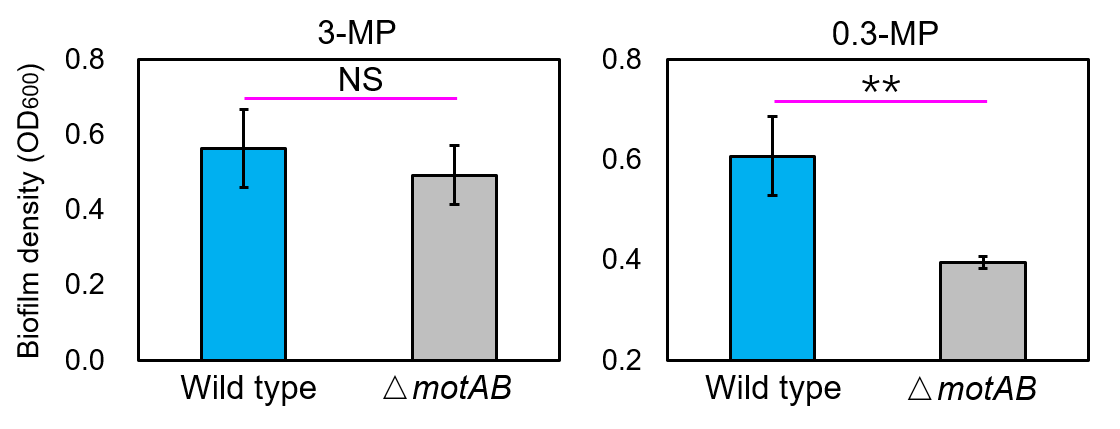


**FIGURE S18** The impact of *motAB* mutation on the colonization of *V. alginolyticus* 2-8 on MPs. The wild-type and the mutant strains were incubated with the 3- or 0.3-MPs for 12 h, followed by cell density measurement. The experiment was conducted in triplicate. ** represents *p*-value < 0.01 while NS indicates non-significance in Student’s t-test.
